# Principles for the design of multicellular engineered living systems

**DOI:** 10.1063/5.0076635

**Published:** 2022-03-02

**Authors:** Onur Aydin, Austin P. Passaro, Ritu Raman, Samantha E. Spellicy, Robert P. Weinberg, Roger D. Kamm, Matthew Sample, George A. Truskey, Jeremiah Zartman, Roy D. Dar, Sebastian Palacios, Jason Wang, Jesse Tordoff, Nuria Montserrat, Rashid Bashir, M. Taher A. Saif, Ron Weiss

**Affiliations:** 1 Department of Mechanical Science and Engineering, University of Illinois at Urbana-Champaign, Urbana, Illinois 61801, USA; 2 Carl R. Woese Institute for Genomic Biology, University of Illinois at Urbana-Champaign, Urbana, Illinois 61801, USA; 3 Regenerative Bioscience Center, University of Georgia, Athens, Georgia 30602, USA; 4 Department of Chemical Engineering, Massachusetts Institute of Technology, Cambridge, Massachusetts 02139, USA; 5 University System of Georgia MD/PhD Program, Medical College of Georgia at Augusta University, Augusta, Georgia 30912, USA; 6 School of Pharmacy, Massachusetts College of Pharmacy and Health Sciences, Boston, Massachusetts 02115, USA; 7 Department of Mechanical Engineering, Massachusetts Institute of Technology, Cambridge, Massachusetts 02139, USA; 8 Department of Biological Engineering, Massachusetts Institute of Technology, Cambridge, Massachusetts 02139, USA; 9 Center for Ethics and Law in the Life Sciences, Leibniz Universität Hannover, 30167 Hannover, Germany; 10 Department of Biomedical Engineering, Duke University, Durham, North Carolina 27708, USA; 11 Department of Chemical and Biomolecular Engineering, University of Notre Dame, Notre Dame, Indiana 46556, USA; 12 Department of Bioengineering, University of Illinois at Urbana-Champaign, Urbana, Illinois 61801, USA; 13 Department of Electrical Engineering and Computer Science, Massachusetts Institute of Technology, Cambridge, Massachusetts, 02139, USA; 14 Computational and Systems Biology Program, Massachusetts Institute of Technology, Cambridge, Massachusetts 02139, USA; 15 Institute for Bioengineering of Catalonia (IBEC), The Barcelona Institute of Science and Technology (BIST), 08028 Barcelona, Spain; 16 Department of Electrical and Computer Engineering, University of Illinois at Urbana-Champaign, Urbana, Illinois 61801, USA

## Abstract

Remarkable progress in bioengineering over the past two decades has enabled the formulation of fundamental design principles for a variety of medical and non-medical applications. These advancements have laid the foundation for building multicellular engineered living systems (M-CELS) from biological parts, forming functional modules integrated into living machines. These cognizant design principles for living systems encompass novel genetic circuit manipulation, self-assembly, cell–cell/matrix communication, and artificial tissues/organs enabled through systems biology, bioinformatics, computational biology, genetic engineering, and microfluidics. Here, we introduce design principles and a blueprint for forward production of robust and standardized M-CELS, which may undergo variable reiterations through the classic design-build-test-debug cycle. This Review provides practical and theoretical frameworks to forward-design, control, and optimize novel M-CELS. Potential applications include biopharmaceuticals, bioreactor factories, biofuels, environmental bioremediation, cellular computing, biohybrid digital technology, and experimental investigations into mechanisms of multicellular organisms normally hidden inside the “black box” of living cells.

## I. INTRODUCTION

**FIG. 1.**
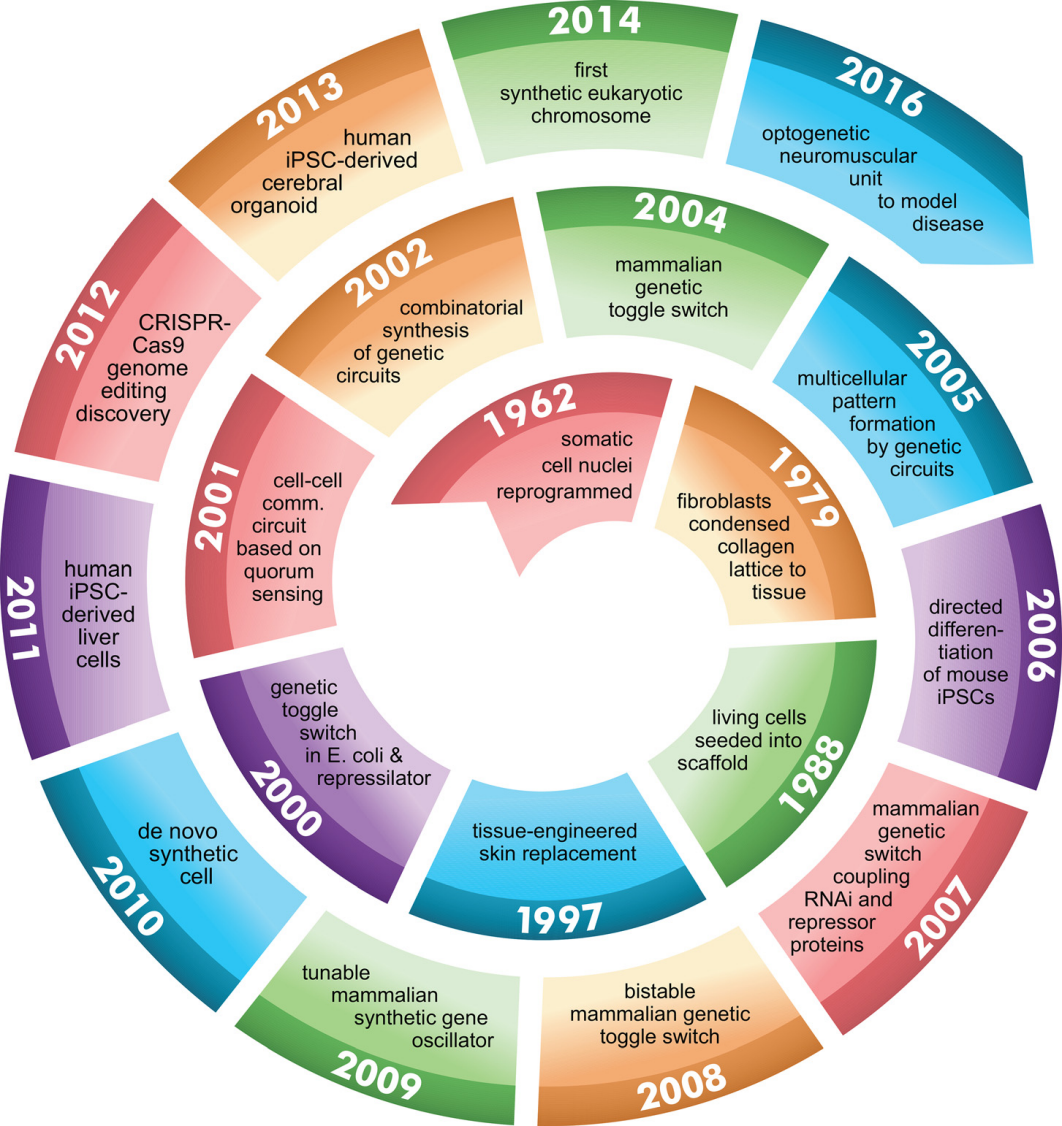
Evolutionary timeline of synthetic biology lying the foundation for M-CELS. (1962) Frog nuclei from intestinal somatic cells are re-programmed to recapitulate embryogenesis into adult frog;^1^ (1964) cracking the triplet codon genetic code;^2^ and (1970) discovery of DNA slicing/dicing with restriction endonucleases;^3^ laid foundation for recombinant DNA and genetic editing (1975) high-performance DNA sequencing;^4^ along with (1978) site-directed mutagenesis provided tools for fine dissection of gene function analysis;^5^ (1979) tissue-like architecture composed of fibroblast-synthesized collagen lattices provided framework for artificial organs;^6^ (1980) DNA cloning resulted in recombinant DNA and the birth of the biotechnology industry;^7^ (1982) discovery of the lambda phage lytic-lysogeny switch generated study of biologic circuits and systems biology;^8^ (1983) PCR amplification yielded working quantities of DNA for genomics from picograms;^8^ (1995) proteins were used as computational elements in living cells;^9^ (1995) electric circuits simulate genetic networks through hybrid modeling;^10^ (1997) artificial skin used to treat severe burn victims;^11^ (1997) Dolly, the sheep, was first mammal cloned from adult somatic cell through nuclear transfer;^12^ (1998) RNAi discovered as a tool for selective gene expression;^13^ (2000) genetic toggle switch designed in *E. coli*;^14^ synthetic oscillatory network designed utilizing cellular transcriptional regulators;^15^ (2001) cell–cell communication effectuates quorum sensing;^16^ (2001) Human Genome Project produces first map of the human genome;^17^ (2002) engineered gene circuits amenable to mathematical modeling;^18^ (2002) engineering and device physics of cellular logic gates;^19^ (2002) combinatorial synthesis of genetic circuits facilitates quantitative analysis of modules and systems;^20^ (2002) chemical synthesis of poliovirus cDNA *de novo*;^21^ (2003) design of genetic circuitry demonstrating oscillatory or toggle switch behavior;^22^ (2003) idempotent vector design for standard assembly of BioBricks;^23^ (2003) engineering the mevalonate pathway for production of terpenoids;^24^ (2004) population control circuit effected through cell–cell communication and quorum sensing;^25^ (2004) design of riboregulators facilitate posttranscriptional regulation of gene expression; (2005) genetically engineered multicellular pattern formation;^26^ (2005) design of light-sensing *E. coli*;^27^ (2006) induction of mouse iPSCs into specialized cells via defined factors;^28^ (2007) universal RNAi-based logic evaluator in mammalian cells;^29^ (2007) tunable mammalian genetic switch coupling repressor proteins with an RNAi target design;^30^ (2008) tunable synthetic gene oscillator;^31^ (2008) synthetic RNA devices for higher-order cellular information processing;^32^ (2009) use of multiplex genome engineering and accelerated evolution for programming cells;^33^ (2009) synthetic bacterial edge detection program;^34^ (2009) synthetic gene networks which count numerically;^17^ (2010) control of bacterial cell by genome chemically synthesized de novo;^35,36^ (2011) differentiation of functional human hepatocytes from iPSCs;^37^ (2011) multicellular computation via genetically encoded NOR gates;^38^ (2011) stripe patterns produced by synthetic genetic circuit;^39^ (2011) metabolic genetic switchboard designed on riboregulators;^40^ (2011) creation of sensing array from coupled biopixels;^41^ (2012) discovery of CRISPR-Cas9 as gene editing tool;^42^ (2013) generation of human cerebral organoids from iPSCs;^43^ (2013) integrating logic and memory with synthetic circuits in living cells;^44^ (2014) complete synthesis of functional eukaryotic chromosome;^45^ (2016) optogenetic control of spinal motorneuron-skeletal muscle contractility unit.^46^

It is now possible to create novel multicellular living machines never seen before. The past few decades have witnessed the convergence of the fields of synthetic biology, bioengineering, stem cell biology, computational biology, and molecular genetics, coupled with increased training of interdisciplinary scientists across two or more of these domains (Fig. 1).^1–46^ This convergence has been accompanied by advances in the design and implementation of technologies such as organoids, microfluidics, biological robots (biobots), nanofabrication, and genetic engineering. The specialized knowledge gained from these interdisciplinary studies now enables the design and engineering of “multi-cellular engineered living systems” (M-CELS) through the combination of all of these technologies. M-CELS represent an engineering approach to building living machines and devices using biological building blocks. They utilize classic engineering modalities of design, modeling, prototype fabrication, testing, and iteration but draw from a toolbox that includes living cells, organelles, proteins, nucleic acids, and biomaterials. The two basic objectives of building living cellular machines are (1) to better understand the properties, mechanisms, and dynamics of these parts in normal living organisms (“to build is to understand”)^47^ and (2) to create better, more intelligent, and biologically efficient machines that can dynamically adapt to their environments in a manner not technically feasible with abiotic machines.

Designing living systems from the ground up requires developing a better understanding of biological building blocks and constructing a set of design principles that define how the blocks fit together and can be manipulated. These design principles enable M-CELS to bring a new perspective to how we think about biology. This fundamental understanding allows M-CELS to be developed for a range of societal applications, including regenerative medicine, agriculture, disease modeling, and pharmaceutics, but these applications currently remain highly challenging experimentally. Biobots (biological robots), for example, have been promoted for use in biomedical applications and pharmacology for developing platforms to gain insights into neural networks and for lightweight compliant actuation using living muscle^48^ and organoids for disease modeling.^49^ These anticipated applications should not be taken for granted but developed in careful engagement with the stakeholders or intended beneficiaries of the particular technology.^50^ The design cycle, in this way, integrates technical, societal, and ethical perspectives and principles, in keeping with the transformative aspirations of the M-CELS field.

Our high-level definition of M-CELS is that these are engineered multicellular systems that have emergent behaviors with desired natural or non-natural form and/or function. This Review provides bioengineers with a set of primary design principles to guide the future design and manufacture of M-CELS. Here, we trace these design principles across three representative M-CELS—(1) organoids, (2) microphysiological systems, and (3) biobots. There are intrinsic and dynamic properties of multicellular systems, which must guide these principles *a priori.* These include spatial and gradient properties that play important roles in developmental biology and create the desired form and function. Such spatial cues guide the development of multicellular organisms by regulating cellular organization and fate, providing the benefit of improved productivity through specialization for organ function. As such, M-CELS are valuable systems for the study of complex emergent behaviors, including intercellular interactions and dynamics. Emergent behavior can be defined as the self-directed, multicellular response occurring as a result of the collective interactions of individual cells between themselves and the extracellular environment that manifests itself by phenomena at the macroscopic systems-level scale.

M-CELS are characterized phenotypically by their overall complexity of form and function and share certain common properties including:

(1) multicellular organization composed of cells with specialized types, functions, and stages of differentiation and maturation;
(2) spatial organization generated through autonomous cell sorting, artificial instructions encoded by bio-macromolecules, and thermodynamically favorable self-assembly;
(3) existence of multiple states characterized by different state variables with various degrees of freedom;
(4) multiple modalities of cell-to-cell interactions, such as, but not limited to, specialized cell–cell junctions, ion channels, humoral receptors, electrochemical channels, cell–cell adhesion molecules, G-protein coupled receptors, and membrane-embedded transducing elements;
(5) macroscale functional modalities (for example, long range contraction, perfusion, metabolic activities, shear fluidic flow, environmental sensing of heat, nutrients, toxins, danger signals, and gravitational and electromagnetic fields).

We present a discussion of the principles for M-CELS' design following an outline based on the typical stages of progression of M-CELS from development to maintenance and adaptation to function. Section II covers the early stages of biological development, focusing on spatiotemporal aspects of cell differentiation, pattern formation via self-organization, stochastic decision-making, and control of cell microenvironment. Section III discusses developmental processes related to the integration of multiple cellular modules (e.g., tissue constructs or organoids) and formation of interfaces, highlighting the roles of reciprocal inductive interactions, compartmentalization and timing, nutrient exchange and transport, and interfacing with abiotic materials. Section IV focuses on maintenance and monitoring of M-CELS, processes of degradation and regeneration, and adaptative responses to external cues and changes in environmental conditions. Section V discusses principles related to the functions of M-CELS, including sensing, information processing, and several modes of actuation (mechanical, chemical, optical, bioelectric, and structural). Finally, in Sec. VI, we present a summary of the framework of design principles presented in Secs. I–V and conclude with an outlook on the future of M-CELS highlighting the need for community-driven efforts and engagement to address potential technical, ethical, and societal challenges.

## II. DEVELOPMENT PART I: EMBRYONIC DEVELOPMENT AND CELL DIFFERENTIATION

To articulate design principles for developing M-CELS, we consider a two-level hierarchical organization framework (Table I). The first level comprises individual subsystems (or modules) whose development is largely self-regulated (that is, these systems do not require feedback from other subsystems to attain primary form and function). The second level involves systems that develop through the integration of multiple modules where inductive interactions between the modules are essential. This hierarchical organization of development is ubiquitous in nature.

**TABLE I.** M-CELS hierarchy ranging from cells (parts and devices) through discrete modular subsystems integrated into complete multi-tissue, multi-organ systems.

	Cell sources	Individual subsystems	Integrated systems
Examples	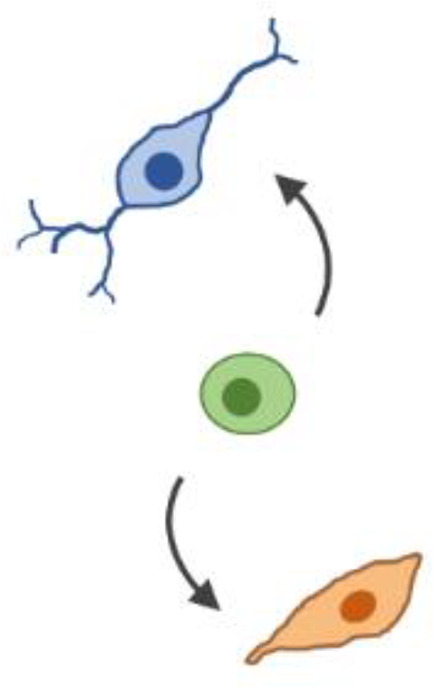 ESCs, iPSCs, adult SCs, primary cells, and cell lines	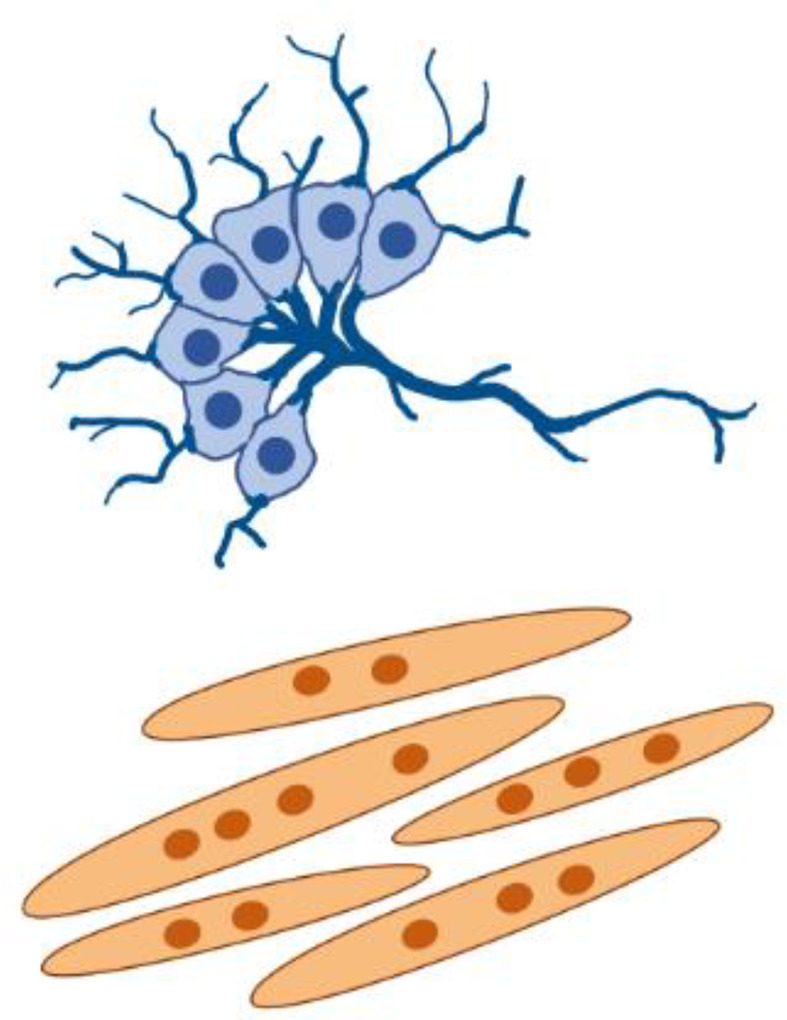 Tissue constructs Neural networks Vascular networks Simple organoid models	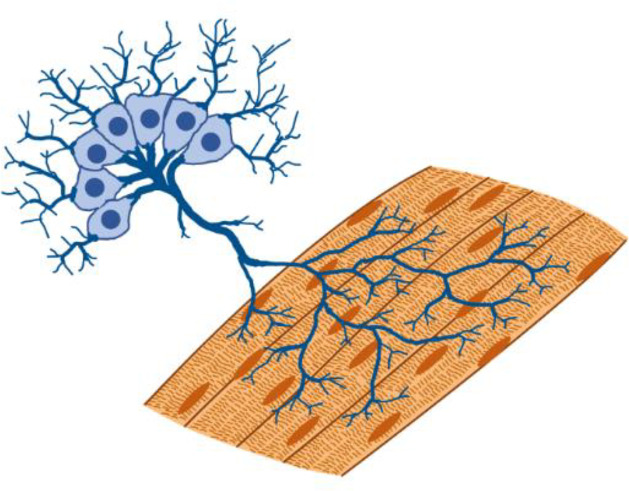 Multi-tissue constructs (e.g., neuromuscular units, myotendinous units) Vascularized tissues and organoids Multi-organoid assemblies
Characteristics	Proliferation capacity Self-renewal	Lower level of hierarchical organization Elementary function Immature phenotype	Higher level of hierarchical organization Complex, coordinated function Mature phenotype
Developmental processes involved	Cell differentiation	Pattern formation Self-organization Stochastic decision-making	Formation of interfaces Reciprocal inductive interactions Activity-dependent adaptation
Corresponding *in vivo* timeline	Very early embryonic	Early to late embryonic	Late embryonic to postnatal

Here, we begin our discussion of M-CELS' design by considering the principles related to autonomous and engineered development of individual modules at the first level of the hierarchical framework. We discuss the developmental tenets essential for driving desired differentiation and organization of M-CELS.

### A. Pattern formation incorporating self-organization

In multicellular organisms, groups of cells working together can adopt different fates and organize themselves in space and time to create complex structures. To make these sophisticated heterogeneous shapes, in early animal development, cell fate is decided based on spatiotemporal patterning, which organisms establish and maintain on their own with few, if any, external guiding cues. If we aim to leverage self-organizing spatial patterning in M-CELS, a starting point is to generalize mechanisms and design principles gleaned from natural developmental systems. To illustrate the success and ongoing progress in this area, we first discuss some fundamental tools of patterning in biological development and then examine the recent advances toward synthetically engineering spatial self-organization.

**FIG. 2.**
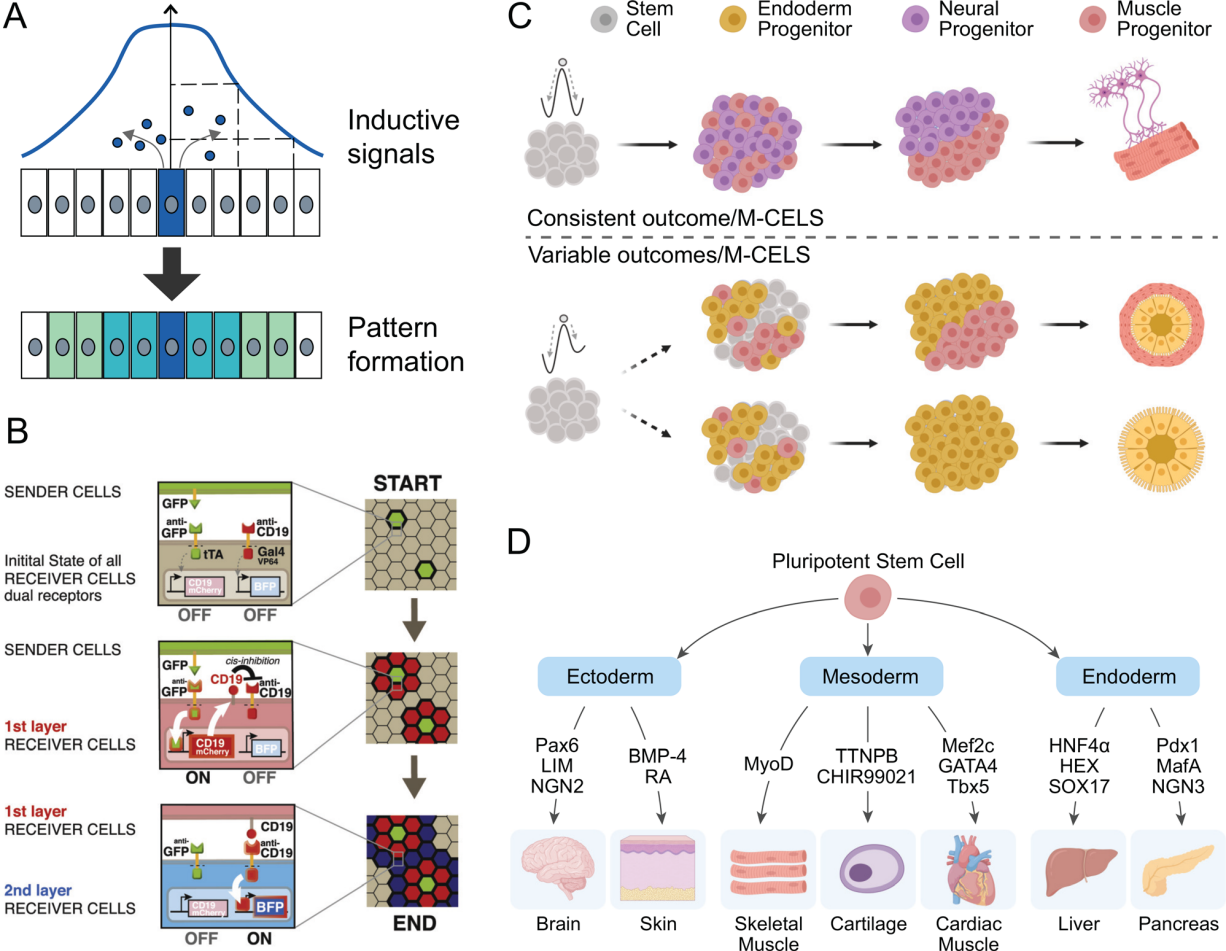
Pattern formation and self-organization in M-CELS. (a) Concentration gradients of morphogens can be generated externally or be formed in the presence of a localized source and are transported across the tissue with cell internalization acting as a sink. These morphogen gradients establish spatial patterns of cell types by regulating developmental programs. This strategy for patterning M-CELS usually results in the definition of a relatively small number of distinct regions. Inductive signals provide the patterning guide for the cells to autonomously self-organize into the relevant functional units. (b) Multiple SynNotch receptors can generate multi-layered self-organizing epithelial patterns. The epithelial layer of sender cells and a clonal population of receiver cells are co-cultivated at 1:50 ratio for 10 (start), 34 (day 1), and 58 h (day 2). Reprinted with permission from Morsut *et al.*, Cell **164**(4), 780–791 (2016). Copyright 2016 Elsevier. (c) The stochastic symmetry-breaking process occurs within the homogeneous cell mass through stochastic activation of genes leading to differentiation and cell specialization yielding cell subpopulations within the larger cell mass. (d) Specific transcription and growth factors can control lineage-specific differentiation from stem cells in M-CELS.

Hierarchically, the cell–cell signaling involved with spatial patterning during development may involve autocrine, paracrine, juxtacrine, intracrine, or endocrine factors. Long-range cell–cell communication is a common tool used to establish spatial patterning in development. A cell can measure and respond to the concentration and duration of a signal that it receives, thereby determining the distance to the source of the signal [Fig. 2(a)]. Using this mechanism, cells acquire their fate based on their relative position in the embryo. Synthetic molecular signal gradients have been demonstrated in mammalian cells using a reconstituted Hedgehog pathway.^51^

Long-range cell–cell communication can also be the basis for complex self-organizing patterns. First proposed by Alan Turing in 1952, Turing patterns are a set of patterns of stripes or spots that can arise from a homogeneous initial condition using only differentially diffusing signaling molecules that activate or repress each other. An example of utilizing Turing-based pattern formation in early stage M-CELS was demonstrated in bacterial populations.^52^ This effort required engineering a slowly diffusing activator and a fast-diffusing inhibitor. A demonstration of long-range pattern formation via Turing-like mechanisms in synthetic mammalian systems is still needed.^53^ The spatial distribution or release of soluble diffusible factors play a key role in generating the radial or longitudinal asymmetric patterns of cellular identities during early embryonic patterning.

Similarly, juxtacrine-like patterning systems have recently been implemented in synthetic living systems. In particular, this effort has included the generation of a synthetic Notch (synNotch) system consisting of the core regulatory domain of Notch, a signaling receptor prolific in animal tissues, linked to a chimeric recognition domain and a chimeric intracellular transcription domain that drive downstream transcriptional outputs.^54^ This tool was used to generate spatial patterns by driving cell–cell sorting through expression of specific cadherin molecules as the downstream signal of synNotch activation [Fig. 2(b)].^55,56^ Cell sorting through differential adhesion has long been known to drive pattern formation in cell aggregates^57^ and is capable of creating an array of different types of structures.^58,59^ Toda *et al.* showed that the combination of synthetic juxtacrine signaling and morphological changes to cell aggregates enabled emergent pattern formation through sequential rounds of patterning of multicellular aggregates, demonstrated by the creation of both two-layer and three-layer structures. It is expected that further engineering of synthetic chimeric transmembrane signaling receptors will enable the fabrication of cell systems with novel morphologies and functions and will also provide a model for testing the dynamics of intercellular interactions in M-CELS.

Geometry also plays a critical role in the outcome of cell fate determination. The control of geometric confinement and size of the system was shown to determine the number of germ layers specified in embryonic stem cell (ESC) cultures.^60^ Going beyond the 2D flat matrix of a Petri dish, 3D printing and embedding cells within soft gel matrices will permit testing of the effects of iatrogenic geometric positioning on the early development of M-CELS.

Finally, patterning of cell populations can rely on purely synthetic solutions as employed by optogenetics or magnetogenetics. Complex patterning of M-CELS will likely need to employ multiple strategies in an iterative fashion, in effect mimicking the developmental programs of natural systems.

### B. Engineering stochastic decision-making

Natural systems have evolved diverse genetic and mechanical mechanisms to process stochasticity during growth and development. Fluctuations in gene expression (i.e., “noise”) are processed by the cell's gene regulatory networks.^61,62^ Gene expression noise can be experimentally minimized,^63–65^ exploited,^66–68^ or ignored. Biomolecular feedback loops are involved in regulating stochasticity in cell growth and patterning.^69^ Mechanical forces, mechanosensing, and mechanotransduction pathways can feedback into the cell and initiate gene expression programs that specify cell fate.^70,71^ M-CELS present an opportunity for interrogating and processing stochasticity in structures composed of multiple cell types and diverse cell-to-cell interactions, which may require a combination of both intra- and inter-cellular approaches. Engineered systems may yield hybrid components that minimize, enhance, or exploit gene expression noise in one parameter regime while ignoring noise in another, such as autoregulation.^61,72^ In M-CELS, stochastic design can orchestrate cell fate determination and cell diversification [Fig. 2(c)].

#### 1. Stochasticity and noise

The ability to engineer stochastic cellular decision making during early development benefits the *de novo* design and synthesis of M-CELS. Despite the highly regulated network for embryonic stem cells' (ESCs) differentiation control by Nanog, Oct4, and Sox2, the expression of Nanog has large cell-to-cell variability that can be tuned through transcriptional noise.^73–75^ Stochastic mechanisms produce diverse cell fates across a cell population or tissue. The use or amplification of stochastic genetic decision making achieves the symmetry-breaking differentiation from an initial homogeneous cell mass into distinct cell populations. Approaches intended to emulate stochastic decision making have led to symmetry breaking from an initially homogeneous cell population, resulting in the generation of distinct cell populations.^76^ This symmetry breaking is needed to produce multiple cell types from one cell type without the use of external cues. Stochastic mechanisms can either generate random patterns of cells with different fates or can act as intermediate steps directed by developmental programs to yield reproducible fates.^77^ In both cases, stochasticity plays a role to diversify cell fates.

Here, we present engineering of stochasticity, cell fate determination, and heterogeneity as a design principle for M-CELS.^78^

One such design principle is the dampening of stochastic fluctuations or noise inherent to biological processes to create more homogeneous and reliable systems. This is needed when cell type decisions require unambiguous outcomes (e.g., liver cell vs neuron) with defined ranges of behavior (e.g., enzymatic activities in the liver vs action potentials). Similar to regulation found among ESCs' transcription factors (TFs) and feedback reported to stabilize viral fate determination,^79^ at least 40% of *E. coli* TFs are enriched with negative autoregulation.^80^ These are likely to increase stability and reduce noise magnitude.^63,79^ Negative feedback also acts as a high pass filter, passing fluctuations with higher frequencies, which can more easily be filtered out by downstream genes in genetic circuit cascades.^61,81,82^

The fundamentals of noise or fluctuations in gene expression have been studied for their sources and consequences.^68,83,84^ Gene expression noise is conventionally quantified by magnitude, frequency, and expression levels^72,85^ and can be measured in mRNA and protein populations. The measurement of additional sources of stochasticity, such as cell growth rates and mechanical stresses, is typically performed using quantitative single-cell microscopy imaging.^86–88^

#### 2. Approaches to modulate biological noise

Noise is a design element that can be actively controlled.^78^ To date, two main approaches have been implemented for controlling gene expression noise and heterogeneity—synthetic biology and pharmaceutical drug treatments. Synthetic gene circuits have been applied as a modular design approach to synthesize artificial tissue and control heterogeneity.^89^ Computational and theoretical analyses of synthetic gene modules for generating population level diversity created a system with increased robustness to uncertain environments. Toda *et al.* engineered mammalian cells to self-organize into multilayered spheroids using synthetic cell–cell signaling.^55^ Specifically, a lateral inhibition gene circuitry provided cross-repression between adhering cells, resulting in cell fate bifurcation. The authors also demonstrate reliable programming of asymmetric or polarized structures by directing subsets of cells to express variable amount of cell adhesion molecules. In another study, engineered bistable gene networks were implemented in yeast to control stochastic and permanent cell fate determination.^90^ By combining synthetic gene regulatory networks with natural regulatory networks, the authors achieve stochastic and irreversible binary cell fate determination. Finally, an overexpressed signaling pulse of GATA6 in polyclonal infected human induced pluripotent stem cells (hiPSCs) provided diversification of differentiated cell types into those found in the three germ layers of the embryo (that is, mesoderm, endoderm, and ectoderm fates).^91^ Additional efforts have synthetically designed noise in gene expression by engineering *cis* regulation at the promoter level^92,93^ or by the optogenetic control of gene expression level and noise of negative feedback gene circuits.^94^

Exogenous drug treatments have been applied to modulate gene expression noise and bias cellular decision making in cell populations without the need to design and integrate synthetic gene circuits. Noise enhancers and suppressors of gene expression from the HIV long terminal repeat (LTR) promoter were screened for, and combinations of noise modulating treatments were shown to provide synergistic reactivation of latent HIV with enhanced control of viral decision making.^95^ Inhibitors of the BAF nucleosome remodeling complex were demonstrated to fine tune noise in the gene expression of the HIV LTR promoter.^96^ Cell-cycle arrest was used to redistribute the cell-cycle distribution of T-cells latently infected with HIV in order to control a cell-cycle and noise dependent viral fate-decision.^97^

Engineering noise in multicellular systems must also account for sources of heterogeneity and morphogenesis that may not directly result from stochastic gene expression. One such example is how morphological and physical constraints maintain homeostasis in the mouse ear epidermis by filtering stochasticity from a layer of randomly differentiating basal cells.^98^ Asymmetric growth has been identified to maintain homeostasis and carefully balance self-renewal and differentiation in stem and progenitor cells.^99^ Another example is stochastic development in the early embryo, which includes coordination of stem cell sorting and positioning along with stochastic gene expression.^100,101^ Early segregation of the mouse blastocyst into epiblast ectoderm and primitive endoderm is sequential with noisy assignment of cell fates occurring prior to cell sorting.^102,103^ Cell sorting has been implicated as a mechanism for patterning and morphogenesis.^100^

Collectively, while continuing to advance the fundamentals of stochastic design and control, our ability to tailor phenotypic diversity by guiding stochastic decision making in M-CELS is achievable. Engineering decision making in M-CELS will require navigating the influence of elaborate intracellular gene regulatory networks, self-organization processes, intercellular lateral forces, and biochemical communication and environmental signaling.

### C. Temporal aspects of differentiation

In attempting to recapitulate *in vivo* development, *in vitro* cultures progress through a temporally defined series of transition states during differentiation [Fig. 2(d)]. Certain early states are universal across cell types through this process, while some diverge in a hierarchical march as they incrementally mature toward lineage specific checkpoints. Evidence for these states can be measured through gene,^104^ protein,^105,106^ and transcription factor^107,108^ expression. Traditionally, these changes have been recorded and observed at the population level. The advent of recent cell specific techniques, such as single-cell RNA sequencing,^109^ as well as accompanying increase in computational power and dimensionality reduction^110,111^ of large data sets, has provided greater resolution into the cell specific dynamics accompanying this temporal window of differentiation. Through these techniques and analysis of multiple stem cell lineages, a universal understanding of this hierarchical march from early to middle to late differentiation has been identified.

While this hierarchical progression seems to be unidirectional *in vivo*, the function of transcription factors as master regulators of these processes has allowed manipulation of these naturally occurring sequences. In this regard, somatic cell phenotypes can be experimentally reverted to more undifferentiated states^112^ as well as across distinct lineages. For example, the emergence of common myeloid progenitor cells from the multipotent progenitor stage depends on a dynamic interplay of UP.1 and GATA.1, which persists to determine lymphoid and myeloid lineage specific differentiation further along myeloid cells terminal differentiation.^113^

Across development, the same gene may have different functions in different organs. Thus, developmental genes greatly reduce the genetic informational load to direct different stages of development. Most of the regulatory factors guiding development can be grouped into two classes: (1) transcription factors and (2) signaling molecules. Broadly, however, following environmental cue or stimulus, transcription factors and accompanying gene units sets the wheels in motion to route undifferentiated cells, whether they be totipotent, pluripotent, multipotent, or unipotent, down a specific lineage path. For example, upregulation of the transcription factor MyoD alone is enough to shift the ultimate cell fate toward myoblast fate because it serves as a master regulator for multiple downstream genes of this lineage.^114^

While general overarching principles for early initial differentiation may exist, such as the need for a stimulus to initiate an active progressive drive toward more and more specialized, honed, and specific cell types, more nuanced and specific alterations also play a major role in determining ultimate cell fate and ultimately M-CELS' health, stability, and function. The level of influence of these activities, as well as the specific genes, proteins, and transcription factors involved, are dependent on cell type starting state of differentiation.

#### 1. Replication and maintenance of pluripotent state

The biophysical microenvironment surrounding the stem cell plays a key role in regulating its pluripotency and cell fate determination into specific cell lineages.^115–118^ Intrinsically, at the genetic level, the pluripotent stem cell state is maintained from the expression of a core of transcription factors, such as Oct4, Sox2, and Nanog, along with the specific methylation state of DNA histones epigenetically. The pluripotent state can be terminated by signals extrinsic to the cells, such as soluble growth factors or the extracellular matrix (ECM).^118^ Growth factors form a major group of extrinsic signals to facilitate or suppress differentiation. While some growth factors promote differentiation, others will maintain the pluripotent state, including transforming growth factor-β (TGFβ) superfamily,^119–121^ fibroblast growth factors (FGF),^122^ and insulin-like growth factor.^123^ It is the balance of intrinsic and extrinsic signals that determine the degree and state of pluripotency-vs-differentiation.

### D. Top-down spatial organization: Microenvironment, compartmentalization

Tissues have well developed spatial organization of cells and ECM that arises from the interaction of morphogen gradients, mechanical cues, and geometry.^124^ Organoids recapitulate some of the spatial organization, but to date, the approach is imperfect. The cells undergo symmetry breaking events and self-organization, and the resulting organoids do exhibit a number of features resembling organs. Without extrinsic cues, however, the overall organoid structure is disorganized, and the structures are heterogeneous in size and shape. Specifically, the overall process can be improved by better controlled conditions that produce consistent initial constraints, control of geometry and morphogen gradients to standardize symmetry breaking events, and dynamic boundary conditions that evolve as the organoids develop.^125,126^ These approaches can be extended to produce functioning M-CELS that model tissues.

Sorting early stage spheroids based on specific criteria, such as size and shape, does improve organoid yield, but other factors need to be considered to further improve yield.^127^ While culture of kidney organoids in a 96- or 384-well format produced more uniform structures, the wide range of kidney and non-kidney cell types present highlights the need for more precise control of the conditions regulating organoid formation.^128^ Other approaches include the presentation of controlled mechanical cues such as the stiffness of the substrate to generate kidney organoids with higher differentiation features highlighting on the possibility to emulate native tissue mechanical properties as a new approach to better control organoid differentiation and function.^126^ A microfluidic device can supply morphogen gradients to control pattern formation during germ line development.^129^ This system provides a very useful tool to examine the role of different features, such as temporal variation of the gradient and cell density, to regulate differentiation.^129^ Organoid differentiation can be improved by culture in bioreactors that provide a well-mixed fluid environment that improves oxygen and nutrient transport.^130,131^

Mechanical properties of the matrix influence the differentiation of organoids in a complex manner.^132^ The different stages of intestinal organoid development require different substrate biomechanical properties. Incorporating a degradable polyethylene glycol (PEG)^132^ created a mechanically dynamic gel that softened over time more closely matching the biomechanical environment needed for growth and differentiation. Other ways to control the environment include the use of hydrogels with light-activated patterns, chemically programmed patterns, or intrinsic control of cellular stochastic processes through synthetic gene circuits as described in Sec. II B.^125,133^ A key challenge to address is how to create dynamic systems that can adapt as the organoid develops that are yet simple enough for high throughput applications.

For various types of M-CELS, spatial organization can be obtained by creating separate microenvironments for different cell types and/or combining with gradients of key signaling molecules to guide interactions. In the fabrication of engineered blood vessels, spatial organization is obtained by extruding endothelium and smooth muscle cells in one annular region and alginate in the outer annular region.^134^ Matrigel attaches to the inner surface of the alginate layer during cross-linking leading to the selective movement of the smooth muscle cells to the alginate interface and endothelium toward the lumen, which forms within a day of fabrication. This vessel structure exhibits a key vessel function, such as contractility and barrier function. While the mechanism has not been established, the authors argue that the vessel structure arises from differential adhesion of the smooth muscle cells to Matrigel and the resulting traction forces.

M-CELS' models of the intestines involve the creation of spatial, chemical, and mechanical gradients in multiple directions. Microphysiological system models have considered the intestinal barrier as separating the acidic intestinal lumen containing microflora from the vascular system. These systems consist of intestinal epithelium in contact with the intestinal lumen contents, extracellular matrix, porous synthetic membrane, and endothelial layer in contact with the culture media mimicking the blood and plasma.^135^ Several key features represent an advance over the simpler Transwell system: flow is introduced into the vascular and intestinal luminal compartment and the membrane is deformable, enabling simulation of peristaltic motion imparting contractile forces on the epithelium and endothelium. Consequently, the epithelial layer begins to develop folds and microvillous-like structures similar to those observed in the intestine. Interestingly, such structures appear with both primary human epithelium and the Caco-2 human colorectal epithelial cell line. Interestingly, these cells formed basal crypts, exhibited microvilli formation, and differentiated into goblet cells, Paneth cells, and enteroendocrine cells found in the intestinal layer.^135^ These systems have been adapted to incorporate cells from human intestinal organoids allowing studies of individual differences in intestinal transport.^136^ Clearly, the presence of mechanical stimuli is critical for differentiation of intestinal cells.

To produce more ordered intestinal structures, Wang *et al.*^137^ used a polydimethylsiloxane (PDMS) mold to create villus structure of the small intestine using micromolded cross-linked collagen on a Transwell membrane. The cross-linked collagen elastic modulus was low enough (∼10 kPa) to enable epithelial cells to form a uniform layer without contracting. To produce a gradient in proliferating and differentiated cells found in intestinal crypts, the authors created gradients of the morphogens Wnt-3A, R-spondin 3, and noggin. Adding the gamma-secretase inhibitor DAPT together with these gradients led to the formation of enterocytes above the highly proliferative cells in the crypt. Collectively, these studies emphasize the importance of matching the substrate mechanical properties with the cell mechanical properties and the importance of morphogen gradients in establishing a variation in differentiation.

## III. DEVELOPMENT PART II: INTEGRATION OF MULTI-CELLULAR MODULES

In the hierarchical organization framework of M-CELS' development, the higher level comprises biological systems whose ontogeny includes the integration of constituent modules. The development of the individual modules is largely autonomous and involves the processes of cell differentiation and spatiotemporal organization discussed in Sec. II.

Integrated systems, on the other hand, rely on bidirectional inductive feedback between the constituent modules for the proper development of a system with a specific form and function. Thus, articulating design principles related to the development of integrated M-CELS requires identifying the key constituents of integrated biological systems, investigating the roles of reciprocal inductive cues between constituent modules and developing methods for modulating or mimicking such cues to engineer the *in vitro* development of integrated systems.

### A. Development of integrated M-CELS through co-culture

An interesting example of an integrated biological system is provided by the model organism *Drosophila* whose larvae exhibit peristaltic crawling motion coordinated by central pattern generating (CPG) neural circuits. When feedback from sensory organs to the central nervous system during development is inhibited, the CPGs still develop normally, and the larvae generate peristaltic movements, however, the locomotion patterns in these larvae are abnormal.^138^ In this example, the CPG is an individual module that develops autonomously through differentiation of neurons and their organization into a circuit that generates patterned output. Similarly, the muscles and sensory organs are separate individual modules. The entire locomotor system, on the other hand, is an example of an integrated system where interactions between CPGs and sensory organs are critical to attain the desired specific function (forward locomotion).

Naturally, a possible approach for developing integrated M-CELS is to identify the key constituents necessary to attain the desired function and establish appropriate co-culture conditions. A successful implementation of this design principle is illustrated by the blood–brain barrier (BBB) model of Campisi *et al.*^139^ While a monoculture of endothelial cells is sufficient to establish a microvascular network, these networks do not have the same physiological properties of the BBB. Campisi *et al.* demonstrated that vascular networks developed by co-culturing endothelial cells with pericytes and astrocytes attain the required levels of barrier permeability. In their work, the authors showed that the vascular surface of the membrane contained a confluent layer of brain microvascular endothelium derived from hiPSCs. This layer exhibited tight junctions and restricted permeability with cells expressing specific transporters for glucose. The neural component contains pericytes, astrocytes and, in some cases, neurons. Pericyte and astrocyte interactions stabilize the endothelium, further reducing permeability and maintaining it for extended periods of time.^140^ The presence of flow in the vascular compartment further stabilizes the endothelium.^137^

Hierarchical development is also observed in the musculoskeletal system where the primary structures of bone, tendon, and muscle (i.e., the constituent modules); each develop autonomously through differentiation, migration, and self-organization of the respective progenitor cells. This is followed by the integration of these preliminary structures and formation of interfaces such as bone eminences and myotendinous junctions between the constituent modules (reviewed by Huang).^141^ Here, the desired function of the system is efficient transmission of contractile forces generated by skeletal muscle to the joints. Engineered muscle-tendon units have been developed by co-culturing tendon fibroblasts and skeletal myoblasts embedded in the ECM in a spatially organized manner. The resulting tissue constructs showed improved tensile and contractile properties.^142,143^

### B. Roles of reciprocal inductive cues

**FIG. 3.**
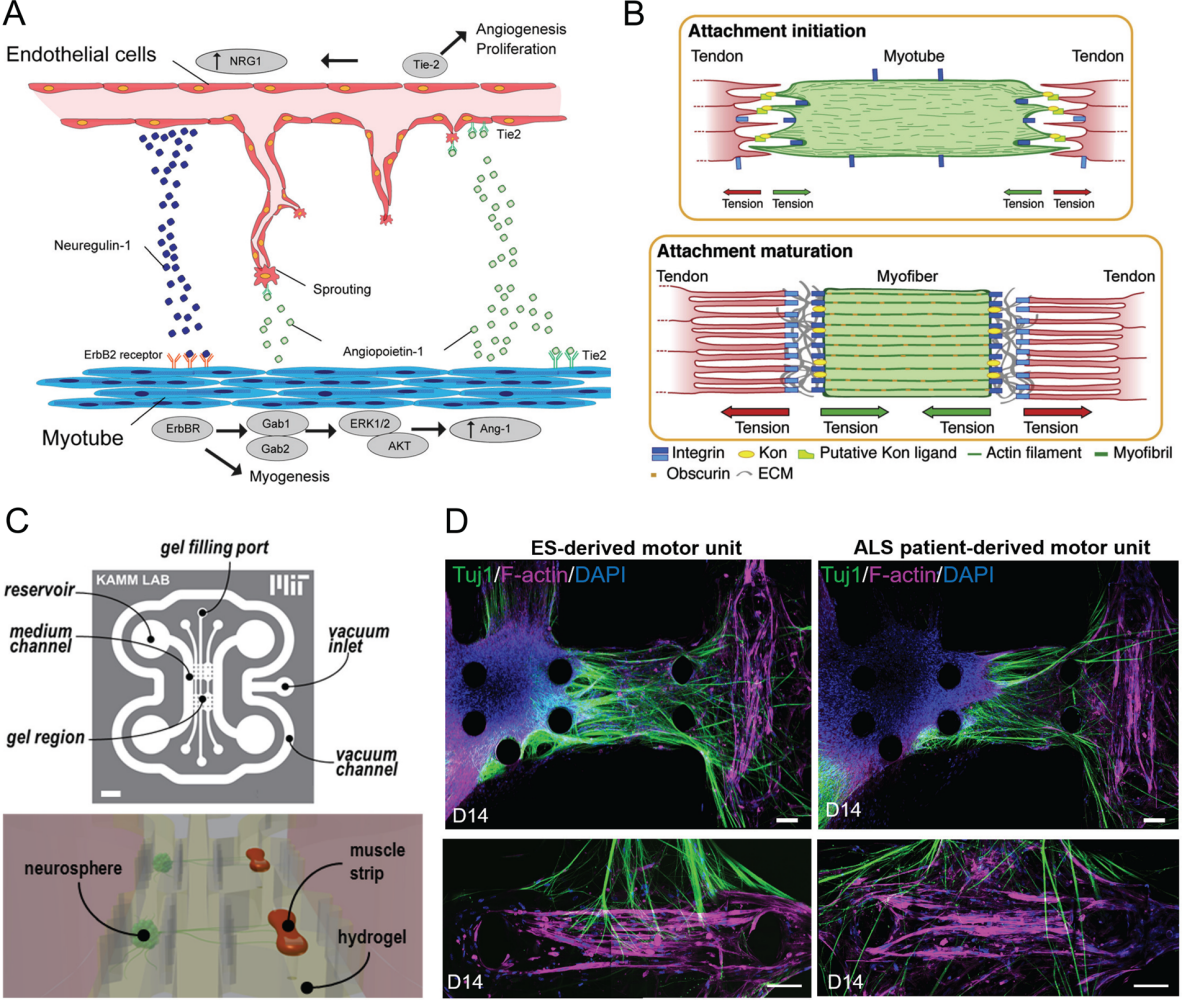
Development of integrated M-CELS involves interactions among multiple cellular modules. (a) Muscle-endothelium co-culture model reveals the roles of reciprocal inductive cues in angiogenesis and myogenesis. Muscle-secreted factors facilitate angiogenic sprouting, and endothelial cell-secreted factors enhance myogenic maturation. Reprinted with permission from Osaki *et al.*, Biomaterials **156**, 65–76 (2018). Copyright 2018 Elsevier. (b) Role of mechanical cues within the developmental timeline of myotendinous junctions. The formation of force-resistant attachments between myotubes and preliminary tendon structures precedes and is necessary for muscle fiber maturation. Reprinted with permission from Weitkunat *et al.*, Current Biol. **24**(7), 705–716 (2014). Copyright 2014 Elsevier. (c) Microfluidic device designs enable compartmentalization of cellular modules. *In vitro* neuromuscular units are developed by housing neurons and muscles in separate microfluidic channels, connected by ECM hydrogel. Reproduced with permission from Uzel *et al.*, Sci. Adv. **2**(8), e1501429 (2016Copyright 2016 Author(s), licensed under a Creative Commons Attribution 4.0 License. (d) Compartmentalized neuromuscular units are used as an *in vitro* model of ALS by developing co-cultures from patient-derived cells. Reproduced with permission from Osaki *et al.*, Sci. Adv. **4**(10), eaat5847 (2018). Copyright 2018 Author(s), licensed under a Creative Commons Attribution 4.0 License.

The development of integrated biological systems involves soluble factor-mediated and activity-dependent bidirectional signaling between the constituent modules. These inductive cues, as well as their physiological outcomes, are critical in the design of integrated M-CELS. Appropriate co-culture systems can serve as models to elucidate mechanisms of interaction and eventually help in developing strategies to modulate or mimic them for M-CELS' design. Osaki *et al.* have recently reported a tissue engineered skeletal muscle-endothelial cell co-culture model where they demonstrated angiogenic sprouting toward muscles and enhanced myogenic differentiation due to synergistic bidirectional signaling mediated by soluble factors secreted by each cell type [Fig. 3(a)].^144^ Oh *et al.* demonstrated that human pluripotent stem cell (hPSC)-derived sympathetic neurons were able to achieve mature phenotype when they were co-cultured with and allowed to innervate ventricular cardiomyocytes, whereas neurons cultured alone remained immature.^145^ Similarly, Martin *et al.* and Aydin *et al.* observed stronger contractions and improved sarcomere assembly in tissue engineered skeletal muscles that were innervated by motor neurons in co-culture compared to muscles cultured alone.^146,147^

The effects of soluble factor-mediated signaling can be recapitulated in M-CELS' development by exogenous supplementation of the factor, thereby allowing phenotypic improvement or maturation without having to rely on co-culture. One approach is to use media conditioned by the target cell type. For instance, skeletal muscle cell-conditioned media, when applied to neuronal cultures, including motor neurons, improves neuron viability, enhances neurite extension, and facilitates development of neural networks.^147^ Similarly, Schwann cell-conditioned media increases spontaneous neurotransmission in developing motor neuron-muscle co-cultures.^148^ If the relevant factors are identified, then it is also possible to supply the pure factor. Agrin, which plays a role in postsynaptic acetylcholine receptor (AChR) clustering in the developing neuromuscular junction (NMJ), can be applied exogenously to induce and/or stabilize AChR clusters.^149^ Agrin and laminin applied in combination can enhance functional NMJ formation.^150^

One must be careful, however, when utilizing exogenous trophic stimulation. Factors that enhance survival and growth may inhibit differentiation and formation of interfaces between cellular modules. Therefore, depending on the relative roles of biochemical cues, exogenous supplementation can impair or facilitate the development of integrated M-CELS. For instance, when *Xenopus* spinal cord neurons were treated with BDNF, GDNF, NT-3, NT-4, Forskolin, IBMX, cAMP, or several combinations of these factors, neurite outgrowth and cell viability were significantly improved. However, this trophic stimulation while keeping cells in the growth state inhibited agrin synthesis in the motor neurons, thus leading to failure of synapse formation when the spinal cord neurons were co-cultured with muscle. Synapse formation was restored when the co-cultures were treated with Schwann cell-conditioned media in addition to the growth factors.^151^

Another important consideration with regard to exogenous biochemical stimulation is that soluble factors typically elicit dose-dependent responses. For example, conditioned extract from muscle tissue enhances neurite outgrowth from motor neurons at lower protein concentrations but inhibits outgrowth at high protein concentrations.^152^ When supplementing cultures with commonly used purified growth factors, the appropriate concentrations are typically documented in the relevant literature. However, it may be necessary when using more complex trophic stimulation, such as conditioned media, to perform a dose-response study to ensure the desired developmental outcomes are achieved.

### C. Temporal aspects of integration

During the development of integrated biological systems *in vivo*, the initial formation, refinement, and maintenance of interfaces between the constituent modules often follow a well-defined time course. Throughout the stages of integration, distinct sets of inductive cues act sequentially to orchestrate the development of interfaces. For example, in NMJ development, muscle secreted FGFs, laminins, and collagen IV chains act as cues that induce differentiation of the nerve terminals. FGF7, FGF10, FGF22, and collagen IV ɑ1/2 chains promote pre-synaptic differentiation in the embryonic stage, β2 laminins are required for the maturation of embryonic synapses during the early postnatal stage, and collagen IV ɑ3–6 chains are necessary for the maintenance of adult synapses.^153^ A corollary of this insight from developmental biology is that failure to recapitulate the proper sequence of inductive interactions *in vitro* may prevent the development of integrated M-CELS. For instance, when developing an M-CELS' model of neuromuscular units using stem cell-derived muscles, if the muscle cells secrete FGFs and collagen IV chains but not β2 laminins, NMJs may initially form but fail to mature since β2 laminins are necessary for their maturation. In such situations, endogenous developmental processes may be restored by several methods, including improvement of stem cell differentiation protocols, genetic engineering, or cell sorting.

Exogenous supplementation of soluble factors can also be a useful tool in the development of integrated M-CELS. It is important, however, to consider the time course of integration and ensure that exogenous supplementation is aligned with the developmental timeline. For example, as discussed in Sec. III B, neurotrophic factors (e.g., BDNF, GDNF, NT-3, etc.) that promote neuron survival and neurite outgrowth can inhibit formation of synapses.^151^ Hence, when developing neuromuscular units *in vitro*, neurotrophic factors can be added during the initial stages of co-culture to improve viability and facilitate axonal outgrowth toward muscles but should be withdrawn once the axons reach the muscle to avoid inhibition of synaptogenesis.

In addition to biochemical signaling, mechanical cues also play key roles in the integration of cellular modules. During the development of myotendinous units *in vivo*, myotubes first extend filopodia toward tendons and initiate attachment. This is followed by an increase in mechanical tension and the subsequent maturation of the muscle fibers and muscle-tendon junctions [Fig. 3(b)]. Interestingly, *in vivo* experiments have shown that the increase in mechanical tension after the initiation of myotube-tendon attachments precedes and is necessary for the maturation of the myotendinous unit. This also means that it is necessary for the initial attachments to be able to sustain mechanical tension.^154^ Thus, recapitulating this developmental timeline in M-CELS' models of myotendinous units requires appropriate design of tissue constructs and culture scaffolds to enable force-resistant attachments between myotubes and tendons. A successful design was illustrated by Larkin *et al.* who first developed tissue engineered tendons with highly aligned collagen fibers and tensile properties similar to that of embryonic stage tendons *in vivo*. The authors then integrated these engineered tendons with muscle cells in a culture scaffold where the resulting tissue bridged between two pins, allowing tension buildup.^143^

### D. Spatial organization: Compartmentalization and microenvironment

In addition to the importance of compartmentalization and microenvironment to cell differentiation (see Sec. II D), these concepts are also vital to the development of integrated M-CELS. Compartmentalized co-culture platform designs may enable successful functional integration of different tissue constructs and/or organoids within a microenvironment that allows for crosstalk and soluble factor delivery. Microfluidic devices are perhaps the most widely used method of spatial organization in an integrated M-CELS design. One example of such an implementation is the neuromuscular unit model of Uzel *et al.*^155^ The authors developed a platform where engineered skeletal muscle tissues and stem cell-derived neurospheres are cultured in individual microfluidic channels separated by an intermediate channel. All channels are then seeded with a continuous ECM hydrogel, which enables the directed growth of axons as well as chemical signaling between neurons and muscles [Fig. 3(c)]. Further developments of this system have enabled *in vitro* modeling and examination of diseases that disrupt or alter the NMJ, such as amyotrophic lateral sclerosis (ALS) [Fig. 3(d)].^156^

**FIG. 4.**
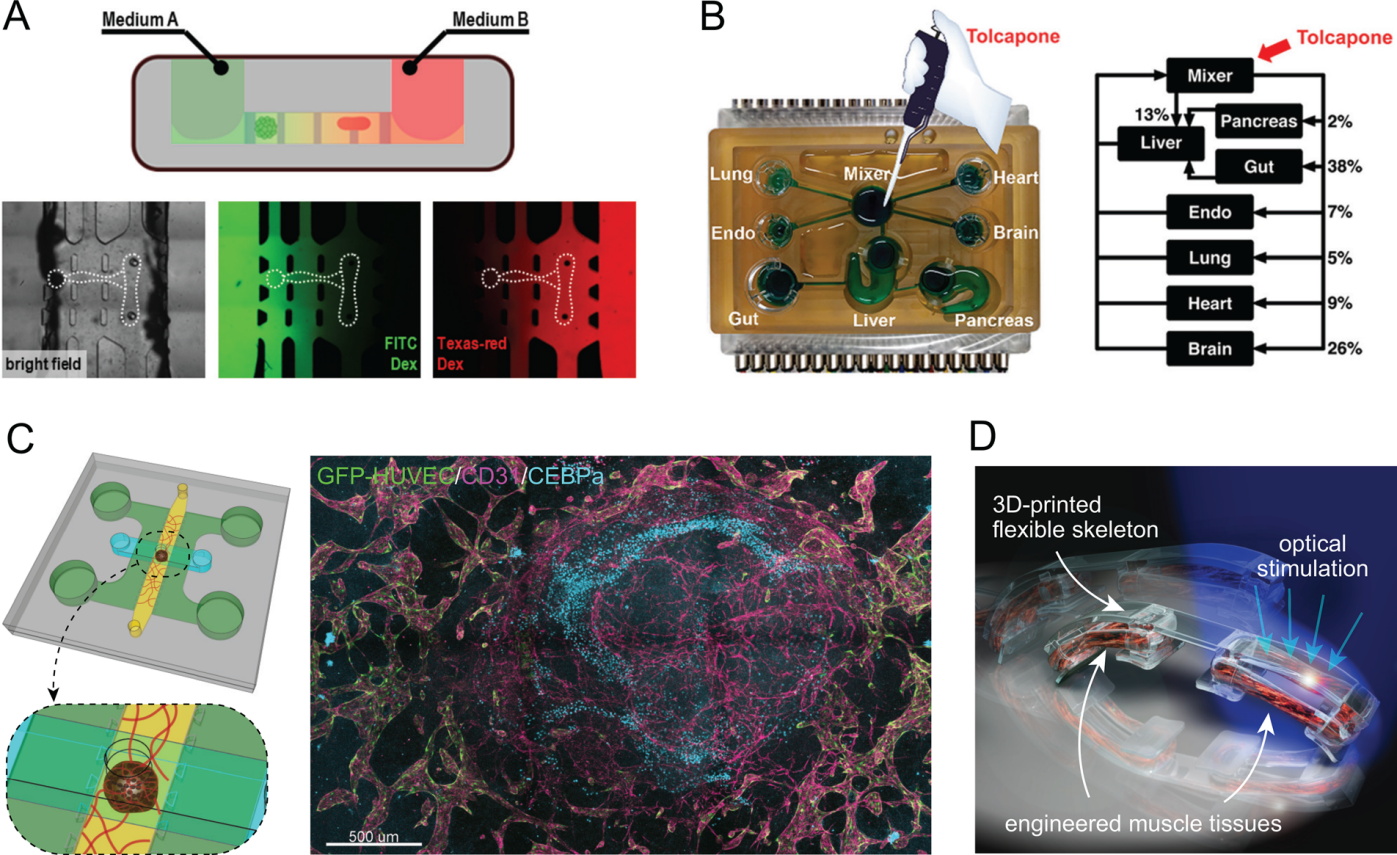
Compartmentalization and microenvironment in M-CELS' designs integrating multiple tissue constructs and organoids. (a) Microfluidic platforms can be designed for the co-culture of different cell and tissue types in controlled microenvironments with separate nutrient delivery to each cell/tissue. Reproduced with permission from Uzel *et al.*, Sci. Adv. **2**(8), e1501429 (2016). Copyright 2016 Author(s), licensed under a Creative Commons Attribution 4.0 License. (b) Microfluidics has also provided a substantial boost for a systems-level understanding of the *in vitro* interaction of diverse organs-on-a-chip, paving the way for the development of a synthetic organism *in toto*. Reprinted with permission from Wang *et al.*, Anal. Chem. **91**(13), 8667–8675 (2019). Copyright 2019 American Chemical Society. (c) Microfluidic platforms may also enable vascularization of organoids, illustrated here by the implementation of a liver organoid embedded within a vascular bed formed by HUVECs and human lung fibroblasts. The immunostaining image shows vessels formed by cells within the liver organoid (magenta alone) integrated with and supported by the vascular network formed by HUVECs (green-magenta colocalization). Unpublished work, images courtesy of Dr. Shun Zhang and Prof. Roger D. Kamm, Massachusetts Institute of Technology, Cambridge, MA. (d) Controlled spatial organization is also possible to achieve on untethered free-standing scaffolds demonstrated here by a biobot design incorporating two separate engineered muscle tissues on a 3D-printed hydrogel scaffold, producing a robotic system capable of multi-directional locomotion. Adapted with permission from Raman *et al.*, Nat. Protoc. **12**(3), 519–533 (2017). Copyright 2017 Springer Nature.

A key advantage of microfluidic devices is the ability to control and manipulate the fluid medium, which can be leveraged to design compartmentalized M-CELS where different tissue constructs are maintained in separate culture media [Fig. 4(a)].^155^ More complex microfluidic platform designs have also enabled the development of organ-on-a-chip models that can capture physiological interactions among multiple organ types and perform metabolomic analysis [Fig. 4(b)].^157^ Furthermore, the ability to control spatial organization and fluid flow may also enable the development of vascularized tissue constructs and organoids within microfluidic platforms [Fig. 4(c)], a long-standing challenge in M-CELS (discussed further in Sec. III E).

While microfluidic platforms allow compartmentalization and control of microenvironment within enclosed channels or chambers, certain applications such as biohybrid robots may aim to achieve spatial organization of multiple tissues on an open and untethered scaffold, therefore requiring alternative methods. One design approach that has been utilized successfully is modular integration of tissue constructs with 3D-printed hydrogel scaffolds. Raman *et al.* have implemented this approach to design biobots powered by multiple muscle tissues capable of multi-directional locomotion [Fig. 4(d)].^158^

### E. Nutrient exchange and transport

Vascularization of tissues and M-CELS is a critical step to surpass scaling limitations imposed by simple diffusion. Diffusion alone is insufficient in providing the necessary nutrient exchange and molecular transport necessary to maintain dense, metabolically active cells within large tissues and M-CELS. Vascularization provides the nutrient exchange network to support cell viability in thick tissues and enables endocrine chemical signaling between large cell systems. By engineering vascularization within M-CELS, the chemical and mechanical cues of an *ex vivo* system can be modulated to recapitulate physiological conditions and provide insight into overcoming the scaling challenges involved during *in vitro* organ generation and design of non-natural MCELS.

An essential element in the design of vasculature is the morphology of the resulting microarchitectures following vasculogenesis. Vasculatures can vary through a broad range of scales with human veins and arteries at around 2 cm, venules and arterioles at 100 *μ*m, and capillaries at around 5–10 *μ*m in diameter. Venules and arterioles are primarily responsible for the rapid transport of nutrients and waste while capillaries provide the surface area for rapid exchanges of nutrients and cellular molecules. In the context of M-CELS, transitions between transport vessels—capillary beds—transport vessels must have careful considerations in its architecture to consider fluid viscosities, flow rates, friction, and burst pressures to ensure that efficient nutrient exchange occurs while the laminar flow remains throughout the system. While designing the microarchitectures of the vasculatures, the bulk mechanical properties of the construct must also be taken into consideration.

To date, *de novo* vascularization has been engineered either in a cell-based or prefabricated network approach. Cell-based approaches involve endothelial cells or progenitor cells undergoing self-organized vasculogenesis within a tissue construct to generate their own extracellular matrix and undergo lumen formation through chemical signaling.^159,160^ This approach comes with the disadvantage that spontaneous vascularization often requires a significant amount of time and an existing mode for the supply of nutrients. Prefabricated network approaches allow for a predefined architecture within a tissue construct prior to the introduction of cells while also providing a mode for delivery of fresh nutrients.^161,162^ However, the design of such networks is limited by the resolution of existing technologies.

### F. Fuzing “living” with “non-living” systems: The biotic–abiotic interface

**FIG. 5.**
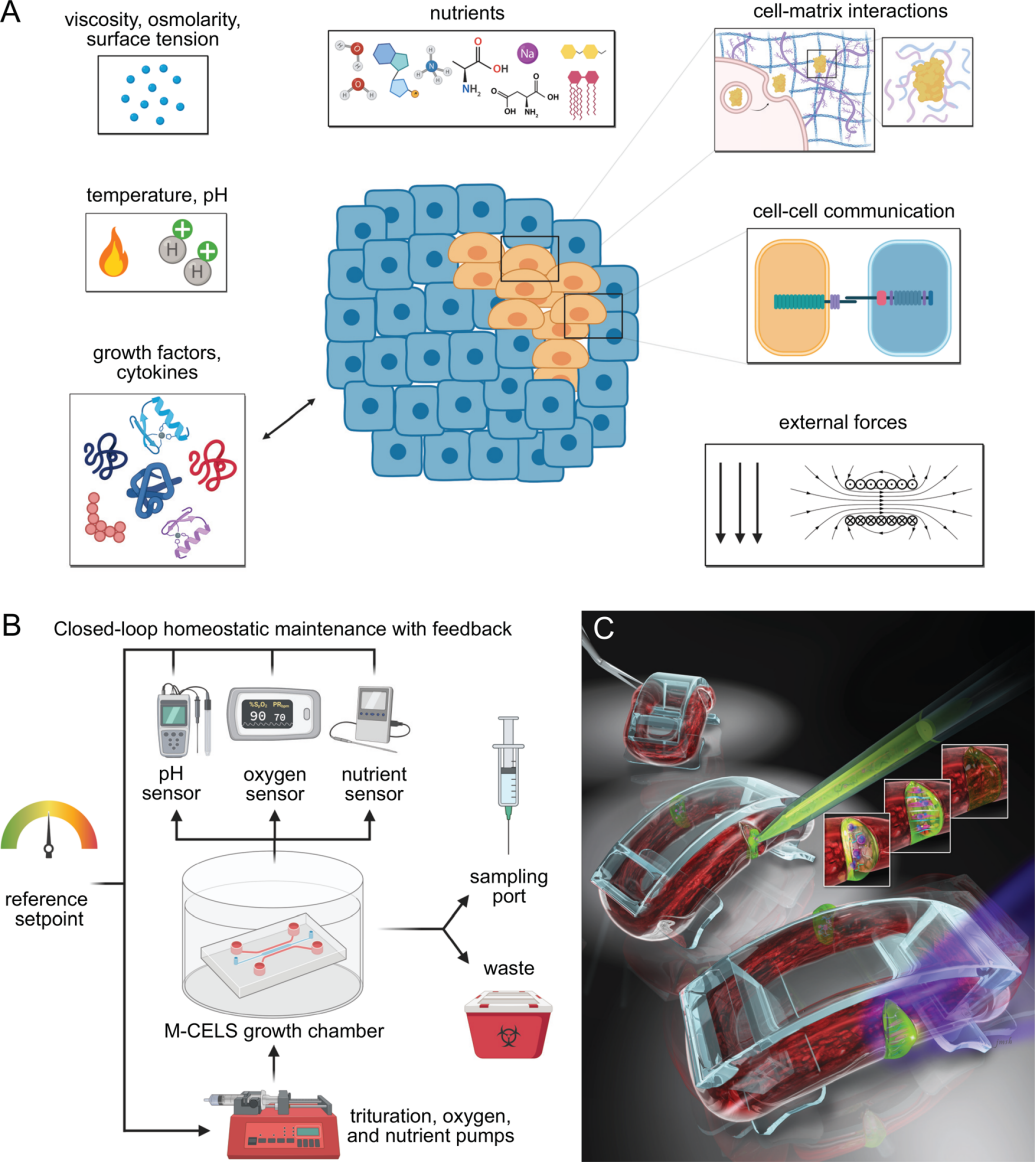
Homeostatic maintenance and engineered repair mechanisms in M-CELS. (a) The cellular microenvironment must enable M-CELS to maintain cell identity, viability, function, and the capacity to respond to various signals that may facilitate their integration into larger systems (nutrients, growth factors, cell–cell communications, and interactions). (b) Homeostatic maintenance of the microenvironment to ensure stability of the M-CELS' phenotype with closed-feedback loops and bioreactor-like control systems. (c) M-CELS must be engineered from stem cells and immune cells to enable self-repair and autonomous response to injury. Adapted with permission from Raman *et al.*, Adv. Healthcare Mater. **6**(12), 1700030 (2017). Copyright 2017 John Wiley and Sons.

This past decade has seen fast-paced innovation in the field of biohybrid systems and molecular machines.^163^ These new systems span the entire spectrum from 90% biologic modules with 10% abiotic material to 90% abiotic material with 10% biologic modules. The state of art of current tissue engineering now allows the fabrication of complex living cellular architectures with chemically synthesized ECM arranged in functional patterns to generate organs-on-a-chip (Fig. 5) or to generate models of tissues such as intestinal tissue, bone marrow, and the blood–brain barrier through 3D bioprinting with living cells.^164^

New neuro-electrode hybrids use nanofabrication (CNTs or graphene) to mimic the ECM of the brain and central neural system (CNS) to promote specific neuronal attachment, limit microglial activation, with flexible geometry for shaping to sub-cellular compartments, promote neurogenesis and synaptogenesis, and enable efficient electrophysiological sensing, stimulation, and recording at the neuronal interface.^165^ A light-sensitive biohybrid flagella-propelled swimmer was designed, comprising a soft scaffold and live skeletal muscle innervated by motor neurons derived from an optogenetic stem cell neural cluster.^166^

A low-power microelectronics controller was embedded in a live jellyfish *Aurelia aurita*, creating a biohybrid robot, and enhancing its propulsive swimming by electrically stimulating muscle contractions at 0.25–1.00 Hz with a square pulse wave (A = 3.7 V, T = 10 ms), thus externally driving its contractions and increasing its peak swimming 2.8-fold.^167^ This demonstrated artificial control of animal locomotion addressing actuation, control, and power requirements in soft robotics. A recent paper describes the history and evolution of these devices from biomimicry through biofabrication to biohybrid systems.^168^

Biohybrid organoids have been created through the three-dimensional assembly of soft, stretchable mesh nanoelectronics throughout the organoid via cell–cell attractive forces from the 2D to 3D tissue configuration during the process of organogenesis.^169^ The stretchable mesh nanoelectronics grew and migrated synchronously with the growth of the 2D cell layers into the 3D organoid structure, showing minimal disruption of cell growth and differentiation. The development of this intimate contact between the cells and associated nanoelectronics will allow tissue-wide electrophysiologic measurements at the single-cell level with millisecond spatiotemporal resolution. These single-cell and spatiotemporal measurements are critical for anatomic-physiologic and developmental studies of the heart and brain.

## IV. MAINTENANCE, DEGRADATION, REGENERATION, AND ADAPTATION

In addition to designing M-CELS that develop, mature, and ultimately function in a desired manner, it is important to ensure that they can maintain consistent and stable phenotypes and functions over extended time periods. Maintenance of M-CELS is perhaps easy to overlook during design and assembly, but it exists on multiple levels from cellular to tissue and organ-scale, and several contexts such as homeostatic, regenerative, and adaptive. Homeostatic maintenance involves ensuring M-CELS exhibit a desired “baseline” phenotype that does not significantly change or degrade over time. Regenerative maintenance consists of repair mechanisms (e.g., inclusion of a stem cell niche that can be stimulated to replenish injured or degraded cells/tissue over time) to give M-CELS the ability to respond to acute injuries and extend the functional lifespan of M-CELS. Finally, adaptation refers to designing M-CELS that can adapt to changes in environmental conditions and/or significant modulations to produce persistent alterations in structure and/or function (e.g., muscle hypertrophy in response to exercise). Incorporation of all of these design principles is vital to successful long-term M-CELS maintenance and function.

### A. Maintenance, degradation, regeneration

On a cellular level, homeostatic M-CELS' maintenance can be thought of as maintaining the appropriate cellular microenvironment, including (but not limited to) temperature, pH, nutrient exchange, ECM deposition/remodeling, and material considerations (i.e., scaffold/device interactions) (see Ref. 170 for a good overview of design principles for 3D cultures and microenvironment) [Fig. 5(a)]. Proper temperature and pH control are relatively simple to achieve with incubators (including incubated microscopes and other equipment) and physiological buffers and can help in ensuring that cells are maintained at ideal and reproducible conditions.^171^ Another solution to maintain steady pH is to use continuous perfusion to replace culture media constantly. These systems, while potentially be difficult to design and implement, have additional benefits of ensuring that cells do not deplete nutrients faster than they can be replenished via intermittent media changes. Perfusion systems have traditionally been used for large-scale cell culture and manufacturing but have recently seen use in microfluidic systems to overcome concerns of impaired cell growth and function due to inadequate nutrient availability.^172–174^ These principles should be applied to M-CELS' design to ensure continuous nutrient availability and ideal pH.

In addition to common nutrients, certain cell types require various growth factors and specific signaling to maintain phenotype-specific genetic programs and homeostasis. Many of these signals are mediated by interactions with, elasticity of, and remodeling of ECM (see Refs. 175 and 176 for summaries of mechanotransduction and matrix remodeling in 3D), which can have pronounced effects on cell phenotype and fate determination.^177,178^ Matrix deposition and remodeling should be considered during M-CELS' design, as artificial matrices or additional cell types (e.g., fibroblasts) may be needed to ensure cells that have the appropriate type(s) of ECM and do not degrade it too quickly (see Ref. 179 for a good review of ECM interactions and challenges with the incorporation of natural and synthetic ECMs). Recently, cysteine cathepsins have been demonstrated to have strong fibrinolytic activity, contributing to the degradation of M-CELS or other engineered tissues containing fibrin.^180–182^ As cathepsins are highly secreted by endothelial cells, this serves as an important consideration especially in vascularized M-CELS or may serve as a target for manipulation of degradation rates; however, dosage optimization is crucial to avoid completely restricting the ability of cells to remodel the surrounding matrix, as these interactions remain important for maintenance of homeostasis and cellular function.

Finally, material characteristics and potential biological interactions must be carefully considered when designing M-CELS. For example, polydimethylsiloxane (PDMS) has been widely used in microfluidic devices due to ease of fabrication, optical properties, flexibility, and permeability; however, it was later discovered that PDMS leaches oligomers into media and absorbs lipophilic factors from media, which can have pronounced effects on cell signaling and cellular phenotypes.^183^ While this is not to say PDMS cannot be used for microfluidic fabrication, these interactions are a prime example of the types of considerations that must be made when choosing materials to ensure that materials are not affecting cellular phenotypes or media composition/nutrient availability.

In the case of acute injury or degeneration, regenerative capacity may be needed, such as harboring stem and progenitor cells in engineered stem cell niches. Stem and progenitor cells are capable of expanding and differentiating into various cell/tissue types; therefore, they could be incorporated into M-CELS as a source for regeneration and repair when necessary. While the conditions for maintaining stem cells in undifferentiated states can vary depending on specific cell types and states, many of the same considerations with the microenvironment apply as above. More specifically, the specific niche environments and ways to engineer these niches have been described and are actively being improved,^184–186^ providing an achievable way to incorporate these cells into a larger M-CELS construct.

On the tissue or organ level, it is important to design M-CELS in a way to protect them from physical injury or to implement solutions to repair them in the event of a physical injury. These solutions may include stem/progenitor cells, as mentioned above, inclusion of immune cells (as macrophages, among others), or a combination of these approaches. Juhas *et al.*^187^ demonstrated the use of stem/progenitor cells for tissue repair using engineered muscle derived from primary cells. The authors first differentiated myogenic satellite cells to create engineered muscle tissue capable of contraction and force generation, as well as harboring a small population of undifferentiated satellite cells, similar to natural muscle tissue. After subjecting this engineered tissue to a cardiotoxin insult, the authors observed satellite cell proliferation and eventual differentiation and replacement of the injured tissue over the following 10 days. Raman *et al.*^188^ demonstrated that similar satellite cell-driven repair of lacerative tears could occur in engineered muscle derived from a cell line [Fig. 5(c)]. Moreover, the pace of repair could be increased through local controlled release of growth factors at the site of damage and exercise of the muscle tissue, resulting in tissue contractility recovery within 2 days. These strategies highlight the potential for stem/progenitor cells to be implemented into M-CELS and replace or regenerate tissue in response to potential damage that may occur over time.

Similar strategies could be applied to other tissues, such as nerve tissue. There have been many studies on peripheral nerve regeneration (reviewed by Ref. 189) utilizing neural (and other) stem/progenitor cells, various scaffolds (both natural and synthetic), and growth factors to improve nerve regeneration after injury. While there are countless combinations of these parameters with varying limitations, many of them have shown promise in improving nerve repair and have significant potential for neuronal repair in M-CELS. Taking these examples together, one can envision how M-CELS incorporating neural and muscle tissues for actuation could be autonomously repaired with the proper stem cell niches and design. A final consideration regarding repair should be considered when choosing materials for M-CELS' design and should a scenario arise where the scaffolds and non-cellular materials comprising M-CELS that need to be repaired or replaced.

Taken together, all of the above aspects require consideration, and potentially engineered control, when designing M-CELS to assure long-term survival and functionality. Maintaining the cell and tissue microenvironment allows for cellular phenotype stability and resistance to degradation, while harboring stem/progenitor cells provide the capability for M-CELS self-repair and regeneration after degradation or injury.

### B. Adaptation

Once M-CELS are maintained for extended periods of time, one of their important properties is that they can adapt to changes in environmental conditions, reflecting the response of organisms and tissues. Such responses represent the functional behavior of the various cell types. For example, an increased force on muscle cells produces hypertrophy to enable the cells to sustain increased loads. These changes can be beneficial, such as in cardiac and skeletal muscle hypertrophy in response to physical stimuli (e.g., exercise), neuronal connections due to learning and conditioning, regeneration and repair after injury, or pathological responses such as heart failure or muscle atrophy through denervation, disuse, or disease.

The adaptive response of skeletal muscle M-CELS to electrical, optical, or mechanical stimulation has been studied extensively.^190^ These stimuli model the effect of various loading conditions that simulate exercise or conditioning. Electrical stimulation models neural stimulation, producing depolarization of the muscle and initiating contraction. Optical stimulation acts downstream to membrane depolarization, opening calcium channels. Mechanical stimulation models contraction and extension, subjecting muscles to strain and forces similar to those encountered *in vivo*. In muscle and other cells/tissues (e.g., endothelial cells), cells respond substantially to various biomechanical forces, including relatively minor/short-term responses (e.g., mechanically gated channel opening) and major/long-term responses (e.g., altered gene expression). Combinations of different modes of stimulations can synergistically produce greater increases in forces than observed with one mode of stimulation alone.^191^ Removal of load stimuli can result in losses of force production and atrophy of engineered muscle, as with native tissue.^192^ M-CELS designed with adaptive responses such as these have functional advantages and are better-suited for long-term survival and ability to perform a wide array of tasks/functions.

### C. Simulating neural activity: The long evolutionary road to cognition, learning, and memory

Attempts to incorporate the neural functions of learning, memory, and cognition have been quite challenging and the M-CELS' cognitive capacity is in its early infancy. Synthetic biologic cognition and memory is still a distant goal, but progress has been made in organoids and biohybrid tissues. The state of art of brain organoid technology has been recently reviewed.^193^ The stem cell-derived self-organized 3D neural aggregates comprising cerebral organoids^194^ have been used as downscaled physiologically relevant *in vitro* models of the human brain^195^ for studies of cognition and neurologic disease.^196,197^ Optogenetic stimulation during neural differentiation can result in permanent changes that extended to the genetic expression of neurons as demonstrated by RNA sequencing.^198^ While cerebral organoids have deficiencies,^199^ such novel or unique neural functions may be useful and applicable for the practical purposes for which M-CELS are designed or built. Recent studies on engram neuronal cells using optogenetic cell labeling and c-fos-promoter transgenic mice are building a foundation for later engineering synthetic neuronal assemblies with long-term memory, a critical attribute for cognition and learning in M-CELS.^200^

Biohybrid neural tissues^201^ facilitate two-way transfer of information between neural tissues and external devices. These devices demonstrate integration with distinct regions of the brain with and multiplexed neural recording.^202^ For example, biofabrication of neural tissue, which forms a three-dimensional neural tissue mimic helps with the assessment of novel neuro-electrodes for the biotic-abiotic interface, and the investigation of neurotransmission and biocompatibility.^203^ Neuromorphic neural interfaces now focus on coupling of biohybrid devices with biological tissue.^204^ Neurobiohybrid swimming machines have been built, which are driven by on-board neuromuscular units and an optogenetic stem cell-derived neural cluster containing motor neurons.^166^

The first biohybrid neural synapses have been implemented through the coupling of an organic neuromorphic device with dopaminergic neurons.^205^ This biohybrid synapse is a leap forward toward the harmonious fusion of biologic neural networks with artificial neuromorphic systems. An earlier biohybrid coupling of neuromorphic and biologic neural networks demonstrated the feasibility of coupling the two networks and implementing control circuits capable of modifying the biohybrid synapse between the neuromorphic and biologic networks.^206^

### D. Noninvasive continuous monitoring of M-CELS

While insightful maintenance of M-CELS is essential for longevity of intended form and function, noninvasive monitoring systems are the crux, which inform maintenance decisions. Without engrained systems for consistent feedback and monitoring of macro- and micro-environments, M-CELS become a black box of unknown perturbations and activity. Therefore, an essential, yet often overlooked principle for designing M-CELS, is inclusion of noninvasive monitoring systems. While engineering concerns, such as cellular components,^207^ scaffolding,^208^ and microenvironment, are critical to M-CELS' design, monitoring and feedback from these engineered construct help ensure longevity of M-CELS as well as garner information for reproducibility and applicability [Fig. 5(b)]. These systems designed to observe, measure, and assess should capture an M-CELS' dynamic progression through multiple phenotypic states without the risk of mechanical, chemical, or physical perturbation of the system. This level of observation will endow a better understanding of the effects timescales and environments have on the phenotypic decisions of these systems. This enhanced understanding eventually could permit greater precision over the development of these systems, as well as allow the heightened knowledge of similarities and differences between the checkpoints of M-CELS compared to *in vivo* developmental systems.

Though multiple monitoring systems and readouts currently exist, a majority of these techniques involve perturbation of the system, which can effectively halt or alter the natural phenotypic progression of M-CELS. Special thought and emphasis should be given to techniques and instruments which allow for continuous or long-term noninvasive monitoring of developing systems. Currently, there exists multiple examples of such monitoring systems that could be easily incorporated in the design schema of M-CELS such as sensor systems,^209^ describing a low-cost sensory system which can measure the optical density of media. These measurements can then be correlated with effective permittivity to ascertain a reasonable noninvasive measure of growth dynamics of a culture. At higher frequencies, this sensory system could be used to monitor changes in the osmolarity capturing minute micro-level changes of M-CELS which would go unnoticed otherwise. All of this information would serve to better understand the micro-environment of the M-CEL system, and specifically, how it behaves on typically overlooked timescales and measures, as well as how it compares to *in vivo* systems and other developmental systems.

While sensor systems provide a noninvasive glimpse into the micro-environment of M-CELS, various imaging techniques have been the preferred method for monitoring systems on macro-level scales. Recent advances, however, have allowed for an even closer look at M-CELS' environments, interactions, and intracellular responses. One such technique is digital holographic microscopy (DHM).^210^ DHM allows for noninvasive monitoring of cell cycle arrest and multiple other parameters such as cell number, confluence, and phase volume. In their study, Miniotis *et al.*^210^ were able to directly measure changes and arrests associated with G1 and G2/M phases via application of specific cell cycle inhibitors. Additionally, spatial light interference microscopy (SLIM) provides label-free assessment and quantification of subcellular biological properties and activity, including dry mass and mass transport.^211^ Currently, it is necessary with this method to first conduct baseline studies to confirm cell cycle phase with more invasive equipment such as flow cytometry. Following confirmation, however, DHM monitoring could provide cell cycle specific information from imaging, alone. Similar monitoring systems can be coupled in parallel with additional imaging systems targeted at various scales, such as macrolevel ultrasound^212^ and magnetic resonance elastography,^213^ which maintain nondestructive imaging structures, yet allow for an even more comprehensive level of understanding of engineering tissues through different phases.

Other noninvasive monitoring systems are possible with genetic manipulation. One common example of this is the use of calcium reporters, which allow for monitoring of functionality in cells and engineered tissues. Juhas *et al.*^187^ in their study described about using endogenous satellite cells to repair engineered muscle tissue, demonstrated this approach by genetically implementing a fluorescent calcium indicator, GCaMP3, which allowed them to noninvasively detect and continuously monitor, in real time, intracellular calcium levels in implanted engineered muscle tissue. By monitoring intracellular calcium levels, the authors were able to monitor contractile function and vascularization via intravital imaging. GCaMP3 and other calcium indicators have also been used to monitor neural activity and are applicable to a wide range of cell types and functions.^214^

Overall, noninvasive monitoring is vital for ensuring that M-CELS are maintained and functioning properly, leading to improved readouts and control of various M-CELS' parameters. The ability to monitor precise cellular processes and macroscale functions can assist with optimization/improvement and inform future M-CELS' design considerations.

## V. FUNCTION

**FIG. 6.**
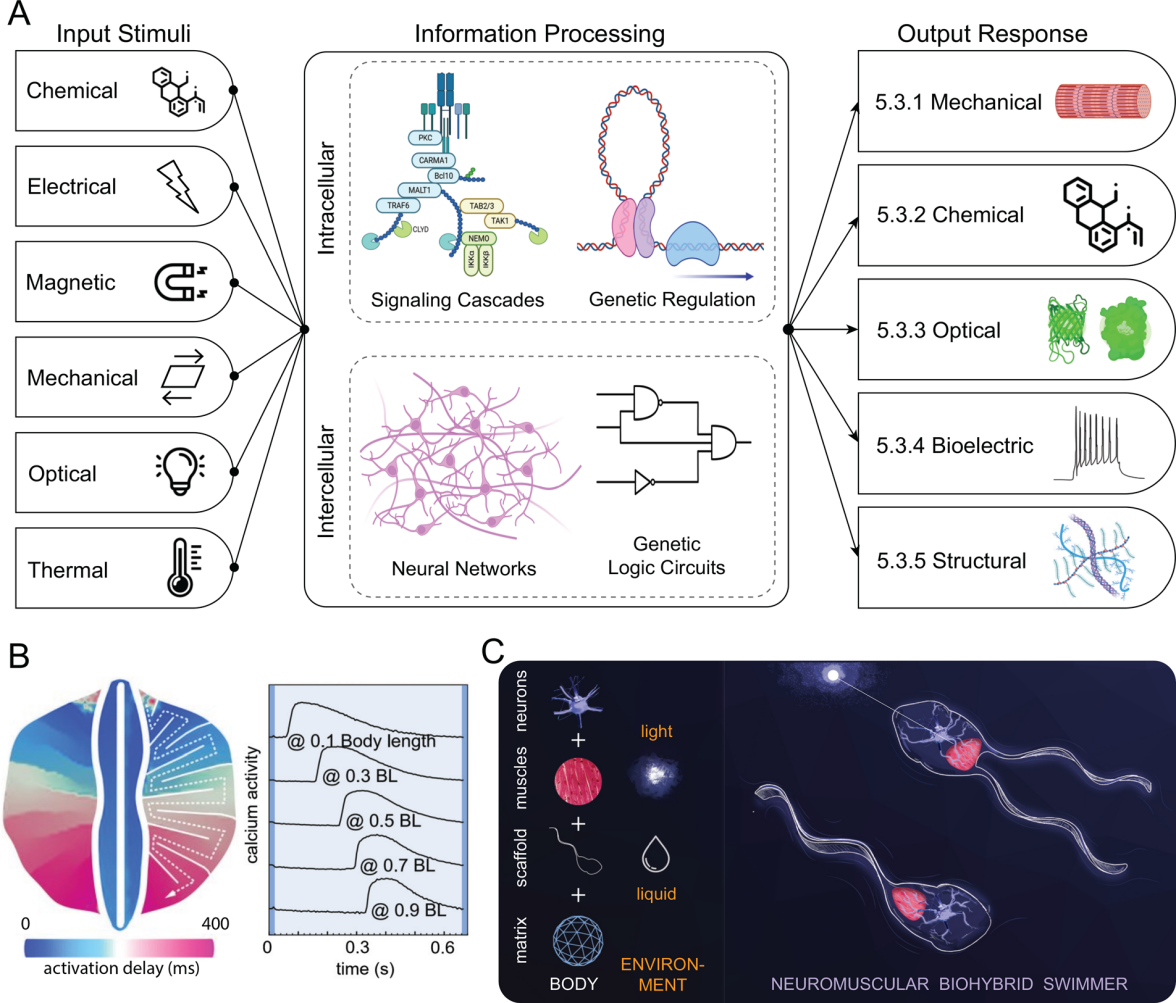
M-CELS integrate and process sensory inputs to generate functional outputs. (a) M-CELS' sensing, processing, and actuation. M-CELS can sense specific extracellular and intracellular stimuli through a variety of sensors. The signals can then be processed to recognize specific levels, patterns, and combinations of stimuli, which, in turn, controls a specific set of output responses in the form of complex biological behavior that can be used for practical applications. (b) Intercellular connectivity of cardiomyocytes arranged in a serpentine pattern on a tissue engineered ray enables a global activity pattern in response to local input. Optical stimulation is applied locally at the front of the fins, and the resulting activation signal propagates via gap junctions through the body length. Republished with permission from Park *et al.*, Science **353**(6295), 158 (2016). Copyright 2016 Author(s), licensed under a Creative Commons Attribution (CC-BY-NC-ND) License. (c) Neuronal actuation and control of muscle-powered biohybrid machines: soft robotic swimmer propelled by flagella, composed of a compliant scaffold, and engineered skeletal muscle tissue innervated by motor neurons derived from optogenetic stem cells. Adapted from Aydin *et al.*, Proc. Natl. Acad. Sci. U. S. A. **116**(40), 19841–19847 (2019). Copyright 2019 Author(s), licensed under a Creative Commons Attribution (CC-BY-NC-ND) License.

A significant part of M-CELS' design is implementation and readout of specific functions. Given the expansive variety of cell and tissue types, materials, and engineering techniques available when designing M-CELS, potential functions can vary considerably, ranging from organoids and platforms to study development to complex multi-tissue assemblies with controllable outputs. Despite the vast conceivable range of functions for M-CELS, the design and implementation of these high-level functions typically involve a combination of three main subfunctions: sensing, information processing, and functional output [Fig. 6(a)].

Signals from the cellular microenvironment and surrounding cells, as well as intracellular signals, serve as the main sources of cellular inputs. As discussed above and in more detail in Sec. V A, cells sense many aspects of their environment, including temperature, pH, soluble factors, matrix composition/mechanics/topography, neighboring cells, and other forces (i.e., shear flow or compression).^215^ Additionally, cells can be engineered to enhance this existing sensory capacity or add entirely new sensing capabilities. All of these sensory inputs and information are vital to detect microenvironmental and external changes but must be processed effectively to inform output and actuation. Additionally, the ability to sense intra- and extracellular changes provides M-CELS with the important capability for feedback regulation and adaptation.

Information processing is vital to the control of cellular functions and outputs both homeostatic and directed. While cell signaling networks can be highly complex, there have been countless studies devoted to elucidating common signaling pathways and their involvement in specific functions at molecular, cellular, and organismal levels. The processing of these and other signals and sensory inputs takes several forms, including relatively quick processing (e.g., phosphorylation and signal transduction cascades) or slow, long-lasting processing (e.g., genetic regulation), combinations of which are important in producing complex and effective responses. In addition to single-cellular processing, M-CELS can also be designed with multicellular processing in mind, such as neural network incorporation.

Finally, actuation and output design allow M-CELS to perform various tasks or provide targeted readouts. Actuation allows M-CELS to carry out high-level functions and includes many possible modalities and types, such as mechanical actuation, chemical synthesis and secretion, optical output, bioelectrical output, and structural deposition or modification. Combinations of these output mechanisms can be employed to design M-CELS with many unique functions for a wide variety of applications, some examples of which are detailed below.

### A. Sensing

#### 1. Extracellular sensing

An immense diversity of mechanisms exists in cells for extracellular sensing. Receptor tyrosine kinase (RTK) cell surface receptors are sensors for many extracellular signals, including growth factors, hormones, trophic factors, and cytokines.^216,217^ In addition, RTKs' cell surface receptors and cytokine receptors are also another important family of receptors that sense cytokines through their extracellular domain.^218,219^ G protein-coupled receptors (GPCRs) are another major family of receptors used for extracellular sensing. This family of integral membrane protein receptors is involved in sensing chemokines, odorants, hormones, pheromones and photons.^220^ Among other sensing modalities and mechanisms, integrins are transmembrane receptors that attach to the extracellular matrix and mediate the response of the cell to the force, rigidity, and ligand distribution in the extracellular matrix.^221^ Ion channel-linked receptors are transmembrane receptors that respond to chemical messengers such as neurotransmitters.^222^ Transient Receptor Potential (TRP) channels are also an important family of ion channels, which use ions for signal transduction and are responsive to several stimuli, including heat, toxins, protons, pressure, and osmolarity.^223,224^

In addition to the sensing mechanisms for extracellular signals discussed, Notch receptors are also able to sense nearby cells.^225^ Immune response pathways also have specialized receptors to sense and interact with nearby cells and pathogens.^226–228^ Nuclear receptors can also sense small molecules that diffuse through the membrane such as steroids and thyroid hormones,^229^ vitamin D,^230^ retinol,^231^ and endocrine-disrupting chemicals.^232^ Synthetic biology also enabled the engineering of sensors for extracellular signals. Examples of engineered sensors include synthetic Notch (synNotch) receptor, chimeric antigen receptors (CAR), engineered light-activated ion channels, and various sensors for environmental monitoring, manufacturing, and health-related applications.^54,233–235^

#### 2. Multi-cellular sensors

Multi-cellular sensing is pervasive in sensory perception and a wide range of mechanisms and specialized multicellular structures exist in living organisms. In olfactory perception, millions of olfactory sensory neurons (OSNs) form a neuroepithelium in vertebrates, which enables the identification of specific odorant molecules using a multicellular combinatorial code.^236^ In auditory perception, hair cells of the basilar membrane act in a coordinated manner to process the frequencies in sound, which makes the perception of complex sound possible.^237^ Moreover, specialized multicellular structures that comprise hair cells in the inner ear are also essential for the sense of balance and movement in mammals.^238^ Proprioception in mammals is achieved through specialized mechanosensory neurons with multicellular diversity in muscles, tendons, and joints.^239^ Visual perception is mediated by the complex multicellular response of photoreceptor cells in the retina, which are capable of phototransduction.^240,241^ Moreover, unicellular organisms can also perform multicellular sensing, such as quorum sensing in bacteria, which enables the detection and response to cell density.^16^

#### 3. Intracellular sensors

Intracellular sensing is vital for every organism, and an immense array of mechanisms both endogenous and engineered exists. Sensing intracellular nutrient levels, metabolic flux through specific pathways and their respective gene expression, *E. coli* will utilize the pathways, which maximize their growth rate, and this can be predicted by an *in silico* model.^242,243^ Contrary to the premise that gene expression pathways evolved solely to maximize biomass production, deletion mutants in *B. subtilis* grew faster than the wild type.^244^ Sensing glucose levels may result in cellular remodeling of nucleoli structure.^245^ Amino acid starvation was observed to increase phosphorylation of nuclear acidic proteins.^246^

Cellular metabolite sensing of bioenergetics for allocation of resources and activation of relevant gene expression is a homeostatic biological function essential for survival.^247,248^ These metabolite sensing and allocation functions are best studied based on mTOR and AMPK signaling. Switching among available biochemical pathways is a critical function during cell growth, proliferation, cell cycle, and stress response, which is facilitated by cell signaling pathways.^249–252^ Early work on metabolite sensing and transcriptional regulation was carried out in *Escherichia coli*.^253^ NAD-sensing was observed in early studies, which revealed that protein synthesis was inhibited by the ADP-ribosylation of elongation factor Ef-2, which is dependent on NAD+.^254–256^ Metabolic intermediates are also sensed through activation of nuclear receptors.^257^

Aside from nutrients and metabolites, there are many other mechanisms for sensing cellular processes and molecules. Epigenetic regulation involves the observation of histone acetylation as key to activating gene expression.^258,259^ Bioenergetic switching from aerobic to anaerobic metabolism is heralded by oxygen-sensing of hypoxia, which leads to epigenetic acetylation of histones promoting lipid synthesis.^260^ Multiple studies have shown that bioenergetic sensing of the cellular energy needs vs fuel levels involves AMPK signaling and mTORC1 signaling as master regulators of cell metabolism. Sensing for intracellular messengers, such as small molecules and ions, is ubiquitous.^261,262^ In addition to the nuclear receptors discussed in Sec. V A 2, another important intracellular receptor is the inositol trisphosphate receptor (InsP3R), which is activated by the signaling molecule inositol trisphosphate (IP3).^263^ Specialized pathways and mechanisms also exist for sensing and regulating proteostasis.^264^ Moreover, pathogen-associated molecular patterns (PAMPs) sensors, damage-associated molecular patterns (DAMPs) sensors, RNA sensors, DNA sensors, and other intracellular sensors for pathogen detection have been observed.^265^

Synthetic biology has enabled the ability to engineer novel intracellular sensors. The first papers in synthetic biology focused on engineering genetic circuits analogous to electronic circuits enabling a genetic toggle switch^14^ and a genetic oscillator.^15^ Promoters and repressors (tet TetR, lac LacI) and transcription factors (lambda phage regulators) served as the prototypical sensors of intracellular conditions to activate these genetically engineered circuits.^26,61,266–268^ Genetic design of metabolic pathways manifest the sensing and regulation of bioenergetics and the carbon cycle, which can be observed in the differential regulation of the lac operon in synthetic *E. coli* where a cost-benefit analysis provides data to parameterize a fitness function for cells as a function of lactose.^253^ Cell-based biosensors have also been engineered for sensing metabolites, small molecules, mRNAs, miRNAs, and proteins.^235,269–271^

### B. Information processing by M-CELS

Cell function arises in a multitude of ways. Many behaviors exhibit straightforward relations between the stimulus and the response. Alternatively, delayed changes in the response to a stimulus that result from changes in cell state (e.g., growth, development, differentiation, apoptosis, and senescence) are activated by signaling pathways that activate or repress genes that control these states. Many of the ligands that initiate these long-term events converge on a limited number of signaling pathways. Furthermore, the same signaling molecules can induce different cell fates due to the presence or absence of other signaling molecules or the state of the cell.^272^ Cells achieve these states using chemical signaling and gene expression, which act like logic gates. Signaling pathways and genetic regulatory elements generate logic gates when the concentration of a key molecule changes dramatically with an input such that the regulatory molecule is either above or below a critical level to activate a response.^273^ These logic gates can be used to create new cellular functions, providing new approaches to regulate the state and function of M-CELS.

Advances in genetic engineering and synthetic biology have led to engineered cells and receptors, allowing for modification or addition of cellular receptors and information processing systems. A high-profile example of this is chimeric antigen receptor (CAR) T-cells, which are being used to treat cancer. Recently, other complex systems are quickly being developed to give cells improved sensory and processing capabilities, such as synthetic Notch (synNotch).^54^ Orthogonal synNotch constructs have been implemented, creating receptor AND-gates, representing improvements in information processing ability, and getting closer to engineering M-CELS with the capability of performing logical computational processing.^274^ The modularity and significant customizability of the synNotch system allows for great flexibility in designing specific responses and functions with control of ligands, receptors, and genes that can be combined in various ways to enable complex cellular information processing. Similarly, genetic Boolean programs^275,276^ represent a powerful way to engineer cells that integrate and process complex inputs, which is also useful for creating cells with desired functions in M-CELS.

In order to improve the specificity of chimeric antigen receptor (CAR) T cell therapy,^277^ created synNotch receptors that acted as AND gates. The extracellular portion of one synNotch domain consisted of the CD19 receptor to enable T cells to recognize CD19 on tumor cells and an intracellular T cell transactivator. On the other synNotch, the CAR mesothelin receptor was inserted on the extracellular portion, which is the same intracellular activation as the CD19 synNotch. The result was a T cell response that was only induced by tumor cells that expressed CD19 and mesothelin and animal studies showed improved survival to tumors expressing CD19.^274^

The concept of AND gates was also used in nanorobots that selectively targeted cell subtypes.^278^ The nanorobot consisted of a DNA origami structure held in place with a clasp with a pair of DNA aptamers. The clasp was released and payload delivered only when both antigens were present. Alternatively, the nanorobot could recognize one target molecule if both aptamers targeted the same molecule. Cell adhesion could be regulated by grafting adhesion peptides or molecules to single-stranded DNA (ssDNA) sequences that can bind to complementary sequences on the substrate.^279^ Addition of a ssDNA without the adhesion motif was used to induce dissociation so that AND, NOT, and OR gates can be produced. These examples show the potential of exploiting logic elements to regulate a variety of cell functions.

### C. Actuation, readout, external communication

#### 1. Mechanical

Mechanical actuation in living organisms serves a vast range of functions from intracellular transport at the subcellular scale to swimming and flying at the organism scale. In animals, muscle tissues are the primary means of generating mechanical output at the multicellular level. Consequently, muscle cells have been used in numerous studies over the past two decades to develop M-CELS capable of mechanical actuation, reviewed in Refs. 48, 190, and 280

Cardiomyocytes are often cultured as adherent cells on a compliant substrate, either as small isolated clusters^281^ or, more commonly, as multicellular confluent cell-sheets,^282–287^ to develop biohybrid machines capable of locomotion or pumping. One of the key features of cardiomyocytes is that they form gap junctions that connect the cytoplasm of neighboring cells, thereby electrically coupling them. In a multicellular sheet of cardiomyocytes, this gap junction-mediated connectivity can be exploited to design complex global actuation patterns triggered by simple local inputs. An example of such a strategy has been demonstrated by Park *et al.* who cultured cardiomyocytes in a serpentine pattern on a compliant substrate. Here, cardiomyocytes at one end of the pattern were stimulated locally and the evoked action potential propagated through the pattern via gap-junction connectivity [Fig. 6(b)]. The resulting undulatory deformation of the compliant substrate was used for locomotion.^285^

Skeletal muscle-based actuation has also been implemented in M-CELS to enable the design of biohybrid walkers,^192^ swimmers,^166^ pumps,^169^ and miniature robotic arms.^288^ In contrast to cardiomyocytes, which are single mononucleated cells capable of sarcomeric contraction, contractile skeletal muscle cells are long multinucleated fibers which form via the fusion of myoblasts. Hence, skeletal muscle bioactuators in M-CELS are developed *de novo* through fusion of myoblasts and the subsequent cytoskeletal maturation of myotubes, often within a reconstituted 3D ECM. The mechanical output of the resulting muscle tissue, therefore, depends on factors that influence this process of development, such as cell source, cell density, ECM type and concentration, culture conditions, and stimulation methods (reviewed in Refs. 289–291). The relationships between these process parameters and the desired mechanical output can be investigated to guide novel actuator designs or improve existing actuators. For example, Pagan-Diaz *et al.* have shown that by differentiating myoblasts into myotubes on a rigid substrate prior to embedding cells in ECM resulted in a threefold increase in the contractile force output compared to actuators that were created by embedding myoblasts directly in ECM.^292^

Effective stimulation and control of muscle contractions are also key considerations for bioactuator design. While spontaneous contractility of muscle cells can be utilized to achieve autonomous function,^281,282^ external stimulation is a more common approach since it offers a means to achieve prescribed contraction dynamics. Cardiac and skeletal muscle cells can be stimulated electrically or optogenetically with precise control of stimulation frequency and intensity. Electrical coupling of cardiomyocytes via gap junctions, as discussed above, enables robust global actuation by local stimulation. Skeletal muscles, however, typically lack this coupling since connexins (gap junction proteins) are downregulated during skeletal muscle development.^293,294^ Evoking coordinated global contractions of a multicellular skeletal muscle bioactuator, therefore, requires effective stimulation of all individual muscle fibers, which can be achieved by appropriate design of tissue constructs and stimulation techniques. In the case of optogenetic stimulation, for instance, the limiting factor is the penetration depth of light. Thus, optogenetic muscle tissues with smaller cross-sectional dimensions can be stimulated more effectively compared to bulkier tissues.^191^ For skeletal muscle-powered M-CELS, neuronal actuation is also possible and has been demonstrated by the development of a biohybrid swimmer driven by on-board tissue engineered neuromuscular units [Fig. 6(c)].^166^

Muscle-based actuation, as it has so far been demonstrated in M-CELS, typically involves the integration of muscle cells with synthetic compliant scaffolds. Scaffold design is, therefore, a significant aspect of M-CELS with mechanical output. Several studies have demonstrated biomimetic scaffold designs which mimic jellyfish,^284^ stingray,^285^ or sperm^281^ morphologies for locomotion. Borrowing from nature in this manner is an effective way to design bioactuator scaffolds, especially when the mechanics of locomotion of the organism, that is, being imitated, is well-understood and the designer can capitalize on existing mathematical models. Conversely, biomimetic M-CELS may also serve as experimental models to study the mechanics of the organism being imitated and possibly help in elucidating design principles which could then be applied to conventional robotics.^295^ Furthermore, it is possible to go beyond biomimetics and develop entirely novel systems using algorithmic design processes. This was recently demonstrated by Kriegman *et al.* who used an evolutionary algorithm to generate, evaluate, and select novel bioactuator designs that achieved desired locomotor performance.^296^

#### 2. Chemical

M-CELS can be engineered to secrete different biochemical moieties in response to a variety of external stimuli or triggers, and this has a wide array of potential applications in medicine, food production, biosecurity, and beyond. Cellular chemical factories are especially interesting in medicine, because they enable personalizing a therapeutic drug dosing regimen to the needs of individual patients. The current standard of clinical care generally involves administering small molecules via ingestion and biologics via injection and often requires consistent and complex dosing schedules that reduce patient comfort and compliance. For diseases with known biomarkers, a multicellular factory could dynamically sense changing biomarkers in the bloodstream and adaptively adjust the production of a therapeutic biologic in a personalized and closed-loop manner. This type of adaptive response could dramatically advance human health, especially when applied to chronic debilitating diseases with unpredictable progression.

One such chronic disease, diabetes, affects over 400 million people worldwide.^297^ Type 1 diabetes is currently treated via regular blood glucose monitoring coupled with insulin injections, a process that is both imprecise and uncomfortable for patients. Insulin is produced by β-cells which reside in clusters within the pancreas known as the islets of Langerhans. The rate at which they secrete insulin is regulated by the level of glucose in the bloodstream, and their loss or dysfunction results in diabetes. Understanding how these cells interact in health and diseased states is critical toward developing and testing new therapeutic interventions for diabetes. As a result, a significant body of research has focused on finding ways to grow and monitor clusters of β-cells *in vitro*.

Since multicellular clusters rely on complex 3D cell–cell and cell–matrix interaction, 2D culture models offer an inadequate representation of the *in vivo* mechanical and biochemical environment.^298^ 2D culture-driven changes in gene expression and phenotype are especially potent in β-cells, which communicate through EphA receptors and EphrinA ligands *in vivo*, and lose their ability to produce insulin in 2D environments *in vitro*. A study by Hammond and colleagues generated a 3D multicellular β-cell construct by growing cells near synthetic “neighbor cells,” microscale gel beads biochemically modified to present EphA and EphrinA as well as a range of other extracellular matrix components derived from decellularized rat pancreas.^299^ Culturing the cells and beads together enabled the cells to interact with each other, and with their synthetic neighbors, while having freedom to migrate. Moreover, void spaces between the cells and beads enabled ready diffusion of oxygen and nutrients throughout the multicellular construct. The researchers showed that this biohybrid multicellular manufacturing method promoted cell survival fivefold and helped in maintaining cell function as assessed by insulin secretion in response to glucose stimulation up to 21 days in culture.

While such *in vitro* culture systems can lend valuable insight into the onset and progression of diabetes, we must eventually find ways to replace lost or dysfunctional cells β-cells clusters in the body to treat this disease. Transplanting β-cells from cadaveric donors is affected by limited supply of donor tissue. Mature β-cells derived from human embryonic stem cells could be a robust and sustainable source for pancreatic cell therapy.^300^ However, both cadaveric and stem cell-based donor cells elicit an immune response from host bodies and, therefore, must be coupled with lifelong immunosuppressive therapy. One way to circumvent this is to encapsulate implanted pancreatic islets in hydrogels that protect the cells from the host's immune system. In the past, this approach has generated a foreign-body response in the host that resulted in fibrotic encapsulation and loss of function of the implant. Promisingly, Anderson and Langer and colleagues have developed a new chemically modified alginate hydrogel that resists implant fibrosis. Stem cell-derived β-cells embedded in modified alginate gel spheres (1.5 mm in diameter) were transplanted in diabetic immunocompetent mice.^301^ The researchers observed that normoglycemia was restored and maintained up to 174 days post-implantation in these mice with very little immune response.

Multicellular chemical factories are promising not only for diabetes, but also for other diseases that can be combated through the administration of biologics. One such example disease is psoriasis, a chronic condition characterized by red and white bumpy scaly patches that erupt on the skin unpredictably. The chronic and unpredictable nature of this disease makes it a prime target for a treatment modality that produces therapeutic proteins on-demand in response to autonomously detected disease biomarkers. Fussenegger and colleagues created such a modality by engineering cells that could detect the proinflammatory cytokines associated with psoriasis, tumor necrosis factor (TNF) and interleukin 22 (IL22).^302^ Importantly, since upregulation of either of these cytokines alone is not specific to psoriasis, they incorporated Boolean AND gate logic into their cellular circuit to ensure that both cytokines would need to be upregulated in order for the cellular factory to be activated. When activated, the cells produced interleukin 4 (IL4) and interleukin 10 (IL10). The researchers encapsulated these cells in an alginate-based hydrogel and tested their functionality when implanted intraperitoneally in a mouse model of psoriasis. Their cell therapy restored normal skin morphology and prevented the onset of psoriatic flares in these mice and, furthermore, was able to detect and respond to TNF and IL22 in human blood samples.

These few examples outline the tremendous power and potential of engineered cellular systems with chemical output response for medical applications. Future studies that develop M-CELS' capablitiy of multi-step decision making in response to mixed external stimuli could lend further depth and diversity to this field.^133^ Applications outside the realm of medicine, such as in producing large quantities of biological molecules for food production, might rely on non-mammalian cells and industrial scale bioreactors.

#### 3. Optical

Several tools have been generated for the rational engineering of optical reporters to provide dynamic readouts of the cellular decision-making processes that take place collectively in M-CELS. These include multi-labeling lineage tracing tools such as Brainbow and Brainbow-type variations.^303–305^ Brainbow constructs utilize recombination events to stochastically express specific combinations of fluorescent proteins (FPs) in cells. This provides a powerful tool for lineage studies where multi-spectral labeling of progenitor cells and their progenies define the extent of clonal expansion of cell types. The number of unique cell types that can be labeled uniquely is constrained by the number of fluorophores that can be independently controlled and clearly separated during imaging. New multi-spectral constructs enable independent switch-like control of fluorescent protein expression within a single construct. For example, the Bitbow system encodes information into the ON/OFF expression state of the fluorescent proteins, with the Bitbow system encoding up to 32 767 unique color states.^306,307^

Fluorescence-based reporters require excitation light sources and these can create issues such as autofluorescence, photobleaching, and phototoxicity. Additionally, light illumination could trigger unwanted stimulation of optogenetic activity. Recently, bright luminescent protein reporters called nano-lanterns have been generated that utilize bioluminescent resonance energy transfer (BRET).^308^ The energy transfer occurs from enhanced *Renilla* luciferase to fluorescence protein. The resulting cyan and orange nano-lanterns are about 20 times brighter than wild type *Renilla* luciferase. Nano-lanterns enable multicolor live imaging of intracellular structures and could be used in conjunction with optogenetic tools to create a palette of optical input and output communication channels between M-CELS and operators.

Multi-spectral optical reporters could be extended to provide optical readouts of more than cell lineage by utilizing genetic switches that are regulated by additional inputs. CaSSA is a platform to create genetic switches using CRISPR/Cas9 (Ca) and DNA repair mechanisms of single-strand annealing (SSA). Multiple gRNAs are expressed in specific patterns to create cell-type manipulations based on the expression of cell-specific genes.^309^ Uniquely addressable switch-like control of fluorophore activity to create multi-spectral digital readouts could facilitate closed control loops that provide optical reporter-based feedback during the dynamic operation of M-CELS. Developing optical-mediated control loops for M-CELS will require the tuning of stimulation (inputs) and outputs (imaging of the optical-based reporters).

#### 4. Bioelectric

Bioelectrical output is another form of output that cells can use for actuation or to report information about their state or the state of their environment. M-CELS incorporating neural networks or organoids could, in theory, use these neural circuits to provide information on their surroundings, especially given recent advances in electrophysiological recording, including microelectrode array (MEA) technology and optical electrophysiology.

While there are a few examples of this functionality in M-CELS currently, it is not difficult to envision cases where M-CELS could be engineered to sense their environment—either internal or external (see Sec. V B)—then respond via neural bioelectrical output or actuation. One example of this functionality would be stress reporting. It is well known that neural circuits exhibit distinct responses under stress conditions *in vivo,*^310,311^ and while the *in vivo* environment is more complex and involves interactions with many different systems and tissues, organoids, and M-CELS are continuously becoming more complex and beginning to model these interactions,^312^ suggesting the capacity to recapitulate these stress responses, as well as other responses to the environment or other signals. In a widely publicized study,^313^ observed oscillatory activity in cerebral organoids that closely mimicked patterns characteristic of the human neonatal brain. These, and similar, studies support the assertion that neural organoids are more accurately representing *in vivo* neural activity and may soon accurately exhibit complex physiological responses (e.g., stress response). As these responses are better understood, they can be monitored and used to observe information on M-CELS' states or engineered for specific downstream actuation.

In addition to neural cells, it is now accepted that non-excitable cells—not just neurons and muscles—sense and generate electrical signals, using bioelectrical signaling to control and coordinate various functions, especially developmental patterning.^314^ While much of the work in this area has been focused on developmental processes and morphogenesis, bioelectrical signaling represents an alternative avenue for engineering and design of M-CELS. With a better understanding, cellular bioelectric properties could be manipulated to control M-CELS' function, providing an additional mode of actuation and output. For example, a pathway involving genetic regulation could be engineered with an upstream bioelectric reporter that could be used to detect pathway activation significantly faster—potentially on the order of seconds—than waiting for genetic changes on the order of hours. This approach would allow for better external readouts on pathway activation and internal states, as well as provide an avenue for the M-CELS itself to sense these states and respond in a specific engineered manner in addition to the inherent response (in this case, gene activation).

#### 5. Structural

Tissues consist of cells within and lining the inner surface of an extracellular matrix that provides a porous structural support and molecular cues that enable cells to adapt to changing conditions. Two key features during development are self-assembly and changes to the microenvironment. Examples include self-organization of endothelial cells and smooth muscle cells to form a vessel *in vitro*^134^ and using the synNotch circuit to promote differential adhesion between cells and produce mulitlayer tissue-like structures.^55^

Expanding the repertoire of molecules in the extracellular matrix can enable programming of M-CELS to synthesize a matrix that meets specific functional needs. The mechanical strength of DNA hydrogels can be adjusted by variation of DNA sequence or creating branching structures.^169^ Aptamers could be synthesized attached to long DNA strands to enable specific binding of proteins or peptides. Single stranded DNA can be synthesized to enable the production of bulk amounts for nanocomposites.^315^ By using metabolic processes to regulate DNA synthesis in the extracellular media, different DNA structures can be produced. By coding the variation of metabolic processes, the DNA structures can undergo locomotion.^315^ Such an approach broadens the way in which extracellular synthesis can be used to create M-CELS for novel applications.

## VI. OUTLOOK

We have outlined the design rules for building multicellular tissues that leverage coordinated communication between component and cells of different types, functions, and stages of maturity. These design principles outline strategies for M-CELS' design, synthesis, maintenance, and modulation and have been discussed in the context of organoid disease models, implantable chemical factories, and biologically powered robots. These emerging applications support the underlying premise of M-CELS by showing themselves capable of self-assembly, homeostatic maintenance, and dynamic programmable adaptation to changing environmental signals. Moreover, they showcase key advances of how sensing and information processing in M-CELS can drive diverse behaviors, such as mechanical, chemical, optical, bioelectrical, and structural output functions. M-CELS with defined output responses, such as actuation, can be engineered via self-organization of groups of stem cells into mechanically functional tissue (Sec. II), top-down defined co-cultures of different cell types (Sec. III), or some combination of these approaches. Illustrative examples of M-CELS that incorporate the various design principles described in this Review are outlined below, representing the first step toward engineering such systems with complex multifunctional behaviors.

Recent advances in neuromuscular organoid synthesis exemplify the design principles outlined in this Review to generate functional, physiological structures via the assembly of separate modalities. Specifically, Andersen *et al.*^316^ generated human cortico-motor assembloids that accurately model the human corticospinal motor tract by combining three parts: human cortical spheroids, human hindbrain/cervical spinal cord spheroids, and human skeletal muscle. To achieve this, the authors first utilize design principles regarding development and cell differentiation (Sec. II) to separately develop these region-specific spheroids. Following this, principles of multi-cellular module integration (Sec. III) are employed to successfully generate corticospinal assembloids, neuromuscular assembloids, and ultimately corticospinal motor assembloids, functionally integrating all three modules. Notably, these assembloids measuring approximately hundreds of micrometers in diameter were able to be maintained and monitored (Sec. IV) for up to 10 weeks in culture post-assembly and demonstrated functional mechanical output (Sec. V) via muscle contraction in response to optogenetic stimulation of cortical spheroids. As the first example of a completely stem-cell derived neuromuscular tissue capable of functional contraction, these assembloids could offer unprecedented insight into human neuromuscular development and disease. Such sub-millimeter scale systems could have potential near-term applications in high-throughput biomarker discovery, drug development, and personalized medicine. Future applications of neuromuscular assembloids as completely cellular and externally controllable implants, such as customizable drug delivery pumps, could open up the use of such M-CELS to new realms. Ultimately, these assembloids—which represent a significant advance in complexity and integration over previous 2D or two-component motor unit models—highlight the promise of the outlined set of design principles for engineering M-CELS with improved applicability.

Certain applications of M-CELS may require larger systems (millimeter-scale and beyond) that also integrate abiotic materials as functional components. Examples of M-CELS in biohybrid robotics highlight how a different fabrication approach can be used to satisfy these parameters. Using many of the design principles outlined in this Review, Raman *et al.* created muscle actuators with the capacity to heal after injury, and a capability that leverages the adaptive nature of M-CELS and has never been observed in abiotic actuators.^188^ First, the authors created biohybrid robots with tissue engineered skeletal muscle (Sec. II) coupled to 3D-printed polymeric scaffolds that helped drive and maintain desired tissue morphology (Sec. III). The muscle actuators were endowed with adaptive response and healing capabilities (Sec. IV) by the inclusion of muscle stem cells that mimicked the satellite cell niche *in vivo* and via targeted stimulation and exercise of specific regions of the tissue. This approach resulted in full recovery of muscle actuator force (Sec. V) within two days of extensive mechanical damage^192^ built on this platform by integrating these muscle actuators with a modular tissue composed of functional motor neurons (Sec. III), leveraging design principles that helped maintain the individual functionalities all the component cells (Sec. IV). These assemblies demonstrated functional mechanical output in response to stimulation of the motor neurons, as with the organoid assembloids described above, showcasing different cell types and design pathways that can lead to M-CELS with similar output responses, depending on the physical size of tissue required and the proposed application. Such systems could be envisioned for use in surgical robotics, such as autonomous muscle-powered grippers that help in performing tasks such as precision suturing. In the longer term, embedding biohybrid robots within exoskeletons could enable deploying them outside of controlled environments, such as in untethered terrain-exploring robots for applications in defense or search and rescue.

While the examples outlined above focus on mechanical actuation, the proposed design principles may be applicable to many other output responses (such as regulated chemical synthesis) and even combinations of output responses. In this respect, their range of uses can be as vast as the imagination of the designer or as numerous as the engineering problems in need of solutions. However, in order to be a useful resource for practitioners of this emerging field, the design principles will require targeted community-driven efforts. Fundamentally, this means refining and updating the principles as new scientific discoveries are made and shared. To do this, the M-CELS' community can mirror other multidisciplinary fields by generating focused symposia to share new information and by creating dedicated training tools and curricula. It can also pursue new methods of engagement by creating databases of design principles or specialized M-CELS' manuscript preprint servers. There has yet to be a collective discussion on how the community can add emerging design principles to the compendium presented in this Review, however, and this remains an important unresolved issue in M-CELS' development and application.

Likewise, to effectively apply M-CELS' technology to solve pressing societal problems, refinement of the design principles will entail thorough integration of ethical and social considerations. As discussed in previous work on the ethics of M-CELS,^50^ this is not a matter of adding ethics onto engineering or pursuing ethics in parallel but rather making implicit value choices more explicit and deciding proactively which avenues of research will have a positive impact. Any M-CELS created, for example, are likely to challenge or complicate traditional notions of biological life and of the human. Prudent choice of cell line (human or non-human), decisions regarding ownership of intellectual property, and careful use of descriptive language (“creature” or “bot”), for example, become especially pressing. Envisioned societal benefits, too, must be subjected to scrutiny to ensure that they are practically feasible—are they still useful when placed in an economic, legal, and cultural context?—and that they represent the actual needs of intended users or beneficiaries. To address these concerns within their work, researchers can draw on the frameworks of “value-sensitive design”^317^ or “responsible research and innovation.”^318^ Thinking beyond the design cycle, it is also crucial that these activities be supported by funding agencies, universities, and other key institutions, such as a special M-CELS' scientific society, funding collaborations with the humanities or social sciences, and motivating broader engagements with policymakers and affected publics.^50^

Looking ahead to the more distant future of M-CELS, these dual challenges (technical and ethical) of curating design principles are not merely obstacles to be overcome. They also represent the transformative aspirations of M-CELS as a field, distinct from pre-existing disciplinary specializations. Researchers have already generated significant collective momentum around this ambitious vision: responsibly design-build-test-debug complex living systems using biological parts. They have crafted new curricula and pedagogical methods for training students in M-CELS^319^ and convened regular interdisciplinary workshops on the ethics and technics of responsible M-CELS research. This community sensibility, ideally, will become a defining and stabilizing feature of the field. Where these trajectories will lead and what they will enable the community to create, however, remain open questions that are worthy of debate. As with the rise of genetics, synthetic biology, and other re-imaginations of biological life, concrete and coordinated community action brings into being new and sometimes unprecedented technological and societal possibilities.

## Data Availability

Data sharing is not applicable to this article as no new data were created or analyzed in this study.
